# CSSQ: a ChIP-seq signal quantifier pipeline

**DOI:** 10.3389/fcell.2023.1167111

**Published:** 2023-05-25

**Authors:** Ashwath Kumar, Michael Y. Hu, Yajun Mei, Yuhong Fan

**Affiliations:** ^1^ School of Biological Sciences, Georgia Institute of Technology, Atlanta, GA, United States; ^2^ Department of Computer Science, Princeton University, Princeton, NJ, United States; ^3^ H. Milton Stewart School of Industrial and Systems Engineering, Georgia Institute of Technology, Atlanta, GA, United States; ^4^ Parker H. Petit Institute for Bioengineering and Bioscience, Georgia Institute of Technology, Atlanta, GA, United States

**Keywords:** ChIP-seq signal quantifier (CSSQ), ChIP-seq, differential binding, epigenetic marks, statistical analysis, k-means clustering, normalization, Gaussian mixture model

## Abstract

Chromatin immunoprecipitation followed by sequencing (ChIP-seq) has revolutionized the studies of epigenomes and the massive increase in ChIP-seq datasets calls for robust and user-friendly computational tools for quantitative ChIP-seq. Quantitative ChIP-seq comparisons have been challenging due to noisiness and variations inherent to ChIP-seq and epigenomes. By employing innovative statistical approaches specially catered to ChIP-seq data distribution and sophisticated simulations along with extensive benchmarking studies, we developed and validated CSSQ as a nimble statistical analysis pipeline capable of differential binding analysis across ChIP-seq datasets with high confidence and sensitivity and low false discovery rate with any defined regions. CSSQ models ChIP-seq data as a finite mixture of Gaussians faithfully that reflects ChIP-seq data distribution. By a combination of Anscombe transformation, *k*-means clustering, estimated maximum normalization, CSSQ minimizes noise and bias from experimental variations. Further, CSSQ utilizes a non-parametric approach and incorporates comparisons under the null hypothesis by unaudited column permutation to perform robust statistical tests to account for fewer replicates of ChIP-seq datasets. In sum, we present CSSQ as a powerful statistical computational pipeline tailored for ChIP-seq data quantitation and a timely addition to the tool kits of differential binding analysis to decipher epigenomes.

## 1 Introduction

Epigenetics causes heritable phenotypes without alterations in the DNA sequence. Histone modifications and chromatin binding proteins are among the most prevalent epigenetic modifications that define epigenomes (Allis and Jenuwein, 2016). ChIP-seq, chromatin immunoprecipitation followed by sequencing, has revolutionized the study of protein-DNA interaction *in vivo,* enabling genome-wide profiling of histone modifications and the localization of chromatin binding proteins (Johnson et al., 2007; Park, 2009). Massive amounts of ChIP-seq data have been generated, illuminating versatile epigenomes that shed light on the mechanisms of epigenetic gene regulation (Mundade et al., 2014; Hollbacher et al., 2020). However, the complexity and variability of ChIP-seq experiments have made quantitative comparisons among ChIP-seq datasets challenging.

In ChIP-seq assay, DNA-protein complexes are immunoprecipitated with antibodies specific for proteins of interest, followed by deep sequencing of the immunoprecipitated DNA. Sequencing reads are aligned to the reference genome. Individual ChIP-seq data has been primarily used to identify DNA regions enriched for the occupancy of chromatin binding proteins or histone modifications within the genome through finding “peaks,” i.e., DNA regions enriched with sequence reads by immunoprecipitation. A number of “peak finding” bioinformatics tools, such as MACS (Zhang et al., 2008) and SICER (Zang et al., 2009), have been developed and benchmarked (Jeon et al., 2020). While identification of peak regions has dramatically increased our understanding of epigenomes, it remains important to capture the rich quantitative metrics of signal intensities that are critical for tracking quantitative and comparative changes in epigenomes among different samples, and the demand for bioinformatics tools that faithfully capture the full expressivity of ChIP-seq data are increasingly (Nakato and Sakata, 2021; Zhao and Chen, 2021). Thus, to harness the full value of ChIP-seq data, it is imperative to develop statistically robust pipelines to expand the tool kits for identification and quantification of differential binding (*DB*) in epigenomes.

Current pipelines for *DB* detection from ChIP-seq datasets can be broadly classified into two groups (Steinhauser et al., 2016; Tu and Shao, 2017; Eder and Grebien, 2022): one group, as exampled in DiffBind (Ross-Innes et al., 2012), ChIPComp (Chen et al., 2015) and *DB*ChIP (Liang and Keles, 2012), utilizes peak-calling algorithms to define peak regions followed by statistical tests to identify *DB* regions among the peak regions; the other group, such as diffReps (Shen et al., 2013), PePr (Zhang et al., 2014), and CSAW (Lun and Smyth, 2016), performs genome-wide analysis to identify all possible *DB* regions. DiffBind and CSAW have been shown to have top performance in their respective categories (Stark and Brown, 2011; Ross-Innes et al., 2012; Lun and Smyth, 2016; Eder and Grebien, 2022). Both DiffBind and CSAW adopt negative binomial models that have been successfully used in popular statistical packages, such as DESeq2 (Love et al., 2014) and edgeR (Robinson et al., 2010), for differential gene expression analysis of RNAseq data. However, due to the overall lower signal/noise ratios of ChIP-seq as compared with RNAseq, compounded by the significant variations of signal intensities and coverage among ChIP-seq datasets by using different protocols, antibodies, or experimental efficiency, extending the statistical methodology developed for RNAseq analysis to ChIP-seq poses challenges. In addition, it is critical for the interpretation of ChIP-seq results to include proper parallel control experiments, such as sequencing of input or non-specific IgG ChIP-seq, for which data distribution does not optimally fit negative binomial model. Further, to maximize the value of ChIP-seq, it is important to detect and quantify differential binding for any designated regions, regardless of peak or non-peak regions. Thus, to develop statistical tools that allow robust comparisons of signal intensities among different ChIP-seq datasets, we must go back to the data and rebuild our modeling choices from scratch.

Here, we have developed a statistically robust pipeline, ChIP-seq Signal Quantifier (CSSQ), uniquely tailored for quantitative analysis of ChIP-seq datasets, capable of comparisons across different experiments for any designated genomic regions. In this pipeline, we adopt a Gaussian mixture model for transformed data instead of directly modeling raw count or discrete data. This method is robust because we first transform count data to continuous data whose distribution can then be approximated arbitrarily well by a finite mixture of Gaussian distributions (McLachlan and Peel, 2000). Specifically, we first process ChIP-seq data using the variance-stabilizing Anscombe transformation (Anscombe, 1948), followed by fitting a Gaussian mixture model through the use of *k*-means clustering, and finally scaling the dataset by estimated maximum value normalization. This approach effectively mitigates background noise and biases associated with individual experimental differences. Such pre-processed ChIP-seq data is implemented for statistical analysis using a non-parametric method suitable for small sample sizes to detect and quantify *DBs*. Benchmarking studies by extensive computational simulations and experimentally validated real ChIP-seq datasets demonstrate the robustness and sensitivity of CSSQ in detection and quantification of *DB*s. In addition to its distinctive features in handling varied signal/noise ratios prevalent in ChIP-seq datasets, CSSQ allows incorporation of input/IgG control datasets and offers superior performance and statistical power with as little as two replicates per group.

## 2 Materials and methods

### 2.1 ChIP-seq datasets, RNA-seq datasets, sequence reads alignment and data preprocessing

Genome aligned BAM files or raw sequence reads FASTQ files of ChIP-seq and RNA-seq datasets (Supplementary Table S1) were downloaded from the source and processed (Consortium, 2011; Shen et al., 2012; Geeven et al., 2015). Raw sequencing reads of FASTQ files were quality checked using FastQC (Andrews, 2022), trimmed using TrimGalore (Martin, 2011; Krueger, 2021).

Trimmed sequence reads from ChIP-seq datasets were aligned to human or mouse genomes (as listed in Supplementary Table S1) using bowtie v1.1.2 (Langmead et al., 2009) to obtain BAM files as described previously (Cao et al., 2013). Aligned BAM files were sorted using SAMtools (Li et al., 2009) and reads within predefined regions were counted using Bedtools (Quinlan and Hall, 2010). For ChIP-seq datasets, both Chromatin Immunoprecipitated-seq data (*IP*) and its control chromatin Input-seq data (*IN*) were counted. The sum of sequence depth normalized counts of each pre-defined region was obtained as their corresponding *IP* and *IN* signals. Background subtracted ChIP-seq signals, as defined as (*IP-IN*), were calculated.

Trimmed sequence reads from RNA-seq were aligned to genomes using STAR aligner (Dobin et al., 2013), quantified using HTSeq (Anders et al., 2015), and analyzed for differential gene expression using the DESeq2 R package (Love et al., 2014). The Ensembl v75 annotation for human genome and RefSeq annotation for mouse genome were used to obtain gene locations and promoter regions.

### 2.2 Anscombe transformation, *k*-means clustering and maximum value normalization

For each pre-defined region, Anscombe transformation, defined by $X^{A}=2\sqrt{X+3/8}$ , where X refers to the pre-processed (*IP-IN*) value, was performed to obtain the respective (*IP-IN*)^A^ values. *k*-means clustering was performed using the function “kmeans ()” in the free statistical software R, with the tuning parameters of *k* centers and nstart = 20, to group (*IP-IN*)^A^ data points into different *k*-means clusters. With selection of *k = 4* clusters, the data points are partitioned into 4 clusters of *low* (*L*), *medium* (*M*), *high* (*H*) and *super* (*S*) signal intensities, respectively. An estimated maximum value *U*, defined as $U_{s}={\bar{x}}_{s}^{A}+3\;S_{s}$ , where ${\bar{x}}_{s}^{A}$ and $s_{s}^{2}$ are the mean and the variance of the cluster with the largest mean (cluster *S*), was used for maximum value normalization to obtain (*IP-IN*)^*^ values by formula (*IP-IN*)^*^ = (*IP-IN*)^A^/U.

### 2.3 Simulated datasets and *DB* induction (*DBI*)

The simulated datasets were generated based on H3K4me3 ChIP-seq datasets from H1 hESCs (GSM733657 and GSM733770 of GSE29611 series). The simulated datasets were generated as follows. The ChIP-seq signals of a real dataset were processed to obtain (*IP-IN*)* signals and served as the base dataset. For each simulation, four statistically similarly simulated datasets were created to have the same data distribution as the base dataset to mimic the null hypothesis of no differences between the datasets, named as *Sim1* ^*#*^ *-Sim4* ^*#*^. Specially, the base (*IP-IN*)^*^ dataset was split into two normal distributions with one covering the “*L”* cluster and the other covering the “*M*,” “*H,”* and “*S”* clusters. The mean and variance of each of these two normal distributions of the base dataset were used to generate simulated (*IP-IN*)^#sim^ datasets from randomly created values that fit into the same data distribution using truncated normal distribution method. The number of data points, the minimum and maximum values of each cluster were maintained for each corresponding cluster of all initial simulated datasets (*Sim1* ^*#*^ *-Sim4* ^*#*^). In addition, randomly picked simulated data points in “L” cluster were converted to 0 to ensure the level of zero inflation maintained as the base dataset from real datasets.

For *DB* induction (*DBI*), data points of randomly selected regions from the 3rd and 4th initial simulated datasets were induced to change values with varying or fixed (2–6) times the SD of the corresponding cluster of the selected regions. In addition, (*IP-IN*)^*sim^ values were constrained between 0 and 1 to avoid outliers. Each simulated dataset was subsequently multiplied by a *Usim*, a value randomly sampled from a Kernel Density Estimate fitted from the estimated maximum values (*U*) of 20 real ChIP-seq datasets, to create corresponding (*IP-IN*)^Asim^ datasets (*Sim1* ^*A*^ *- Sim4* ^*A*^), followed by reversed Anscombe transformation to derive corresponding simulated *(IP-IN)* ^*sim*^ datasets (*Sim1-Sim4*). For each simulation condition, a total of 300 simulation runs were performed on 300 independently generated sets of simulation datasets with 1 run/simulation dataset (of *Sim1-4* datasets).

### 2.4 Differential binding analysis

*DB* analysis using DiffBind, CSAW, and CSSQ were performed as follows.

***DiffBind* v2.8.0** (Ross-Innes et al., 2012): Aligned bam files of ChIP-seq datasets and the coordinates of the regions of interest in bed format were fed to DiffBind for analysis. “*db*a.count,” “*db*a.contrast” and “*db*a.analyze” functions were used to perform differential binding analysis. For these functions, the “minMembers” parameter was set to 2 to indicate the number of replicates, and the method was set to use DESeq2 available within DiffBind. An FDR cutoff of 0.05 was used to identify significant *DB* regions. For simulated datasets, *DBA* objects were created from (*IP-IN*)^sim^ values, followed by *DB* analysis.

***CSAW* v1.14.0** (Lun and Smyth, 2014; Lun and Smyth, 2016): Aligned bam files of ChIP-seq datasets and the coordinates of the regions of interest in bed format were fed into CSAW for analysis. The “regionCount,” “windowCounts,” “filterWindowsGlobal,” and “normOffsets” functions within CSAW were used to quantify and normalize signal over regions of interest. To fit the quasi-likelihood model and perform statistical tests, CSAW uses the “asDGEList,” “estimateDisp,” “glmQLFit,” “glmQLFTest,” “mergeWindows,” and “combineTests” functions. An FDR of 0.05 was used to identify significant *DB* regions. For simulated datasets, “RangedSummarizedExperiment” objects were created from (*IP-IN*)^sim^ values, followed by *DB* analysis.

***CSSQ***: Aligned bam files were used to quantify the number of reads that overlap the regions of interest using Bedtools (Quinlan and Hall, 2010). Depth normalized *IP-IN* signals were subsequently derived by subtraction of normalized read counts of ChIP-seq sample by that of its input-seq sample for each of the predefined regions. All negative *IP-IN* values were converted to 0. This pre-processed *IP-IN* data points were fed to CSSQ for Anscombe transformation, normalization, *k*-means clustering and *DB* analysis using a non-parametric statistical test. Regions that had *IP-IN* values above zero in one or more datasets were kept for subsequent analysis. An FDR cutoff of 0.05 was used to filter for significant *DBs*.

### 2.5 Hierarchical clustering, metagene analysis and signal distribution plots

Hierarchical clustering of regions was performed using MeV (Howe et al., 2011) and Metagene analyses were performed using GenPlay genome analyzer and browser (Lajugie and Bouhassira, 2011; Lajugie et al., 2015). The signal intensity of 100bp sliding windows covering the entire defined region were plotted. Aligned bam files were used to quantify the number of reads in each window for each sequencing dataset. The counts for each dataset were normalized to 10 million mappable reads. *IP-IN* signals were subsequently derived by subtraction of normalized read counts of ChIP-seq (*IP*) dataset by that of its corresponding input-seq (*IN*) dataset for each window.

## 3 Results

### 3.1 The CSSQ analysis pipeline

#### 3.1.1 ChIP-seq data pre-processing, transformation, k-means clustering and normalization

**FIGURE 1:**
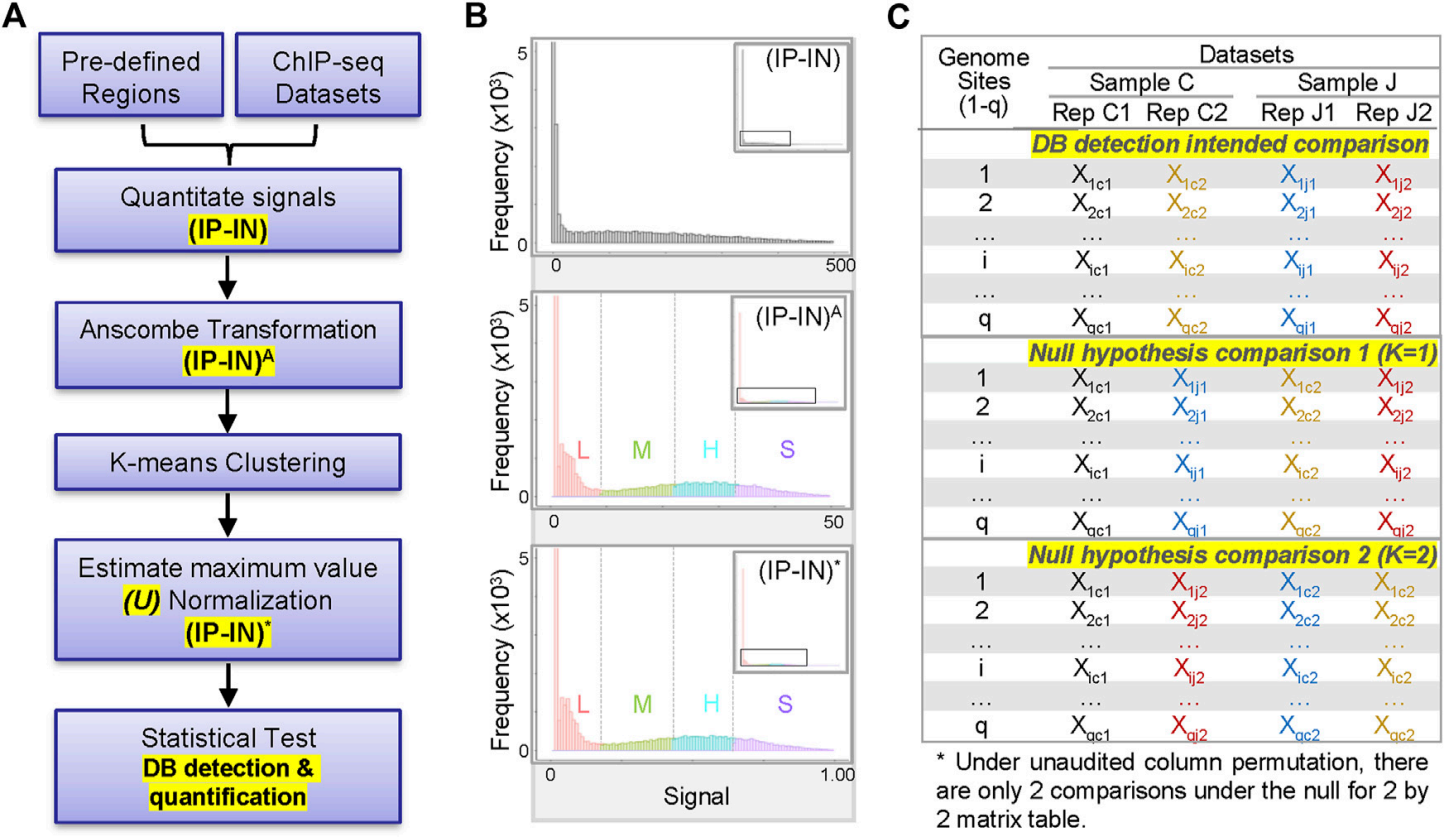
Overview of CSSQ pipeline. **(A)** Flow chart of CSSQ pipeline. **(B)** Representative histograms of H3K4me3 ChIP-seq datasets throughout CSSQ pipeline. Zoomed out histograms are shown as insets. (IP-IN)^A^: Anscombe transformed data. (IP-IN)*: CSSQ normalized values. L, M, H, S clusters are derived from *k*-means clustering and color coded as pink, green, blue, and purple, respectively. **(C)** Representative matrix table of datasets for *DB* detection using CSSQ.

CSSQ integrates data pre-processing, transformation, and parameter estimation in a Gaussian mixture model (clustering, normalization) and statistical test, enabling vigorous *DB* detection and quantification (Figure 1A). To develop a statistically robust pipeline, we first evaluated the data distribution of representative ChIP-seq signals (Figure 1B, Supplementary Figure S1). For sample ChIP-seq datasets of H3K4me3, an active histone mark enriched at active gene promoters (Barski et al., 2007; Heintzman et al., 2007; Jambhekar et al., 2019), the sum of sequence depth normalized counts covering a 2-kb promoter region centered around transcription start site (TSS) of each gene was calculated for H3K4me3 ChIP-seq data (*IP*) and its control chromatin Input-seq data (*IN*) to obtain their corresponding *IP* and *IN* signals. Negative *IP-IN* values that reflected regions with signals below background noise were converted to 0 to enhance data visualization and to facilitate downstream statistical transformations. The overall data distribution of *IP-IN* exhibited similar patterns to *IP*, with high concentration of values around 0 followed by a wide range of data points with mixed multi-modal distribution patterns (Figure 1B, Supplementary Figure S1).

To have a better model on the distribution of complex data for optimal statistical pipeline development, we fit a mixture model instead of a single distribution. To be more specific, we transformed the raw, discrete ChIP-seq count data to continuous values so that statistical analysis using Gaussian mixture distributions is feasible. Among the various widely used transformation approaches for transformation of non-Gaussian to Gaussian data or Gaussian mixture models, based on our extensive numerical experience, we chose the Anscombe transformation for its variance stabilizing properties and suitability for both small and large values (Anscombe, 1948). The Anscombe transformation is defined by $X^{A}=2\sqrt{X+3/8}$ where the constant 3/8 is introduced to stabilize the variance of the transformed data X^A^; this constant is negligible if the X itself is large. Intuitively, the Anscombe transformation of (*IP-IN*) signals into (*IP-IN*)^A^ values effectively increased the data distribution differences, so that the resulting continuous distribution data can be approximated arbitrarily well by a finite mixture of Gaussian distributions (Figure 1B).

**FIGURE 2:**
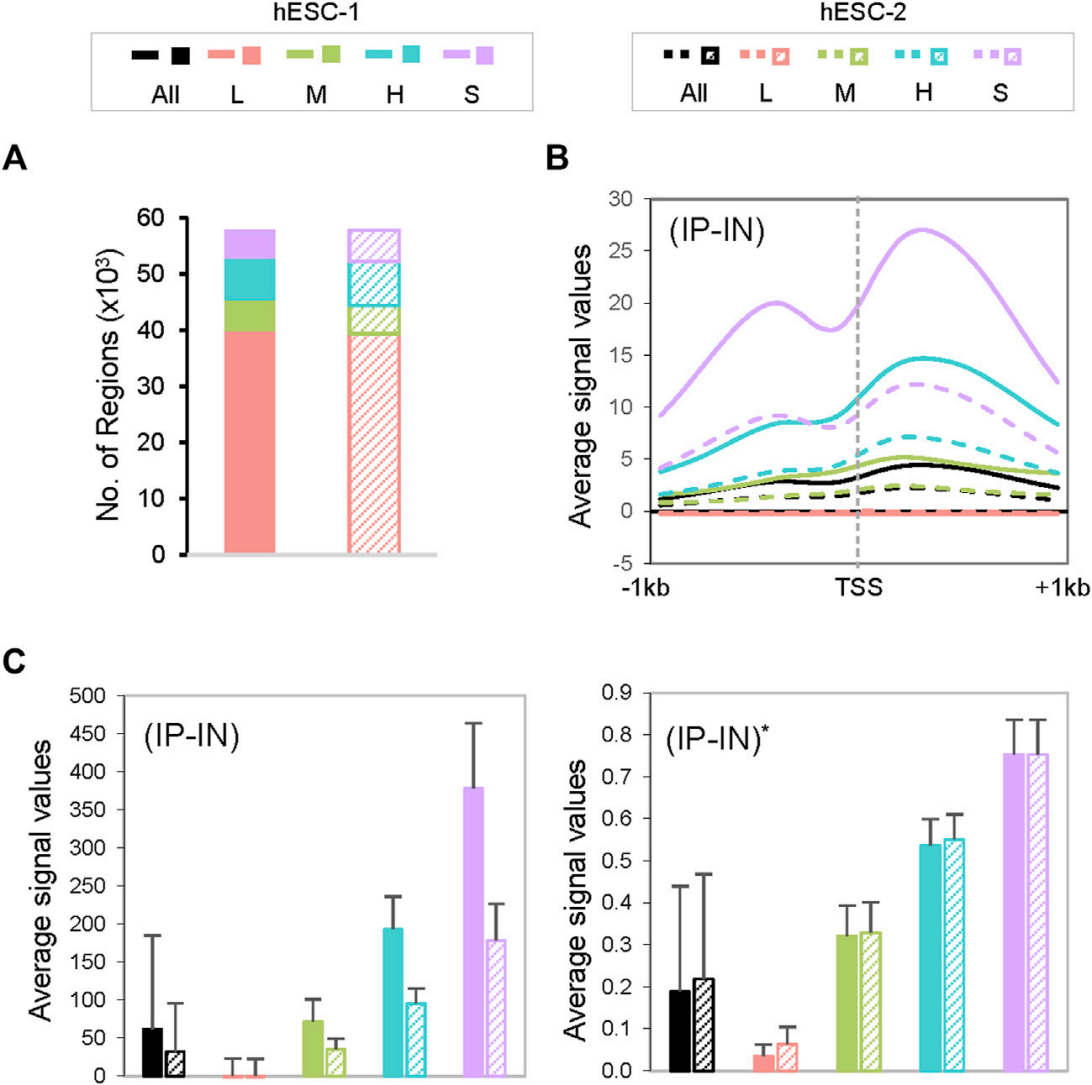
Characterization of CSSQ clusters. **(A)** Cluster allocations of datapoints of two representative datasets of H3K4me3 ChIP-seq of hESCs**. (B)** Metagene analysis of H3K4me3 ChIP-seq signals centered around transcription start sites (TSS). **(C)** Bar charts of mean ChIP-seq signal values pre- (left) and post- (right) CSSQ normalization. Error bars: standard deviation.

Next, we estimated the parameters of the Gaussian mixture model through *k*-means clustering (McLachlan and Peel, 2000) with the goal of normalizing disparate datasets to an equal scale via an estimated maximum normalization approach. This is crucial for *DB* identification and quantification across datasets because individual ChIP-seq datasets often differ substantially in their data distribution and range of signal intensities, even for replicate datasets from the same biological sample (Figure 1B, Figure 2, Supplementary Figure S1). To robustly compute the estimated maximum value (designated as “*U”* factor), we first utilized the *k*-means clustering algorithm to partition data points into *k* = 4 clusters representing categories of *low* (*L*), *medium* (*M*), *high* (*H*) and *super* (*S*) signal intensities (Figure 1B, Figure 2). Data points within each *k* cluster have minimal in-cluster variances and thus are considered as within the same Gaussian distribution. The mean and variance of each cluster were calculated to estimate the parameters of the corresponding components in the Gaussian mixture model. The cluster with the largest mean (*S* cluster) was used to derive the value of *U*, defined as $U={\bar{x}}_{S}^{A}+3s_{S}$ , where ${\bar{x}}_{S}^{A}$ and $s_{s}$ are mean and standard deviation (*SD*) of the *S* cluster. Calculated U values from real ChIP-seq datasets indicate a wide range and variations among different datasets (Supplementary Figure S2). All (*IP-IN*)^A^ values were subsequently normalized to obtain corresponding (*IP-IN*)^*^ values, defined as (*IP-IN*)^*^ =(*IP-IN*)^A^/*U* for each data point. This step effectively mitigates the disparity of signal levels between datasets and minimizes experimental bias between replicates (Figure 2, and Supplementary Figure S3). We should highlight that *k = 4* is optimal for balancing the goodness-of-fit and model complexity of all datasets we tested.

#### 3.1.2 CSSQ DB analysis

To detect and quantify *DB*s, CSSQ uses a statistical test based on the Welch’s two-sample *t*-statistic for data points of each row/region. The difference between the comparisons was calculated as follows:

$$T_{i}^{\left(obs\right)}=\frac{{\bar{X}}_{ij}^{*}-{\bar{X}}_{ic}^{*}}{\sqrt{\frac{\sigma_{ij}^{2}}{n_{j}}+\frac{\sigma_{ic}^{2}}{n_{c}}}}$$

where j and c are the two samples being compared and ${\bar{X}}_{ij}^{*}$ and ${\bar{X}}_{ic}^{*}$ are the means of the *i*-th row for sample *J* and *C*, respectively (Figure 1C). When $n_{j}$ and $n_{c}$ , which represent the number of replicates in each sample, are moderately large, one may use the sample variances to estimate the variances $\sigma_{ic}^{2}$ and $\sigma_{ij}^{2}$ for the *i*-th region in the two samples. However, most ChIP-seq datasets have few replicates, e.g., $n_{c}=2$ , $n_{j}=2$ , and such small numbers prevent making good estimates of $\sigma_{ic}^{2}$ and $\sigma_{ij}^{2}$ . To circumvent this issue, we used a novel approach by taking advantage of information from column-wise *k*-means clustering of each dataset and estimating the *i*-th rows $\sigma_{ic}^{2}$ and $\sigma_{ij}^{2}$ using the average variances of their corresponding clusters from their respective dataset. For instance, for a given i -th row, say we have an observation $X_{ij1}$ that belongs to the “*M*” cluster of the *J1* column/dataset, and the observation $X_{ij2}$ that belongs to the “*H*” cluster of the *J2* column/dataset (Figure 1C). We estimate $\sigma_{ij}^{2}=\left(s_{mj1}^{2}+S_{hj2}^{2}\right)/2,$ where $S_{mj1}^{2}$ and $S_{hj2}^{2}$ denote the variances of *M* cluster of *J1* column/dataset and *H* cluster of *J2* column/dataset, respectively.

Given the *q* observed test statistics $T_{i}^{\left(obs\right)}$ ’s where *i = 1,2…q and q = number of regions*, it is intuitive to declare *DB*s if the corresponding *p*-value is statistically significant. To obtain the *p*-values, CSSQ uses a non-parametric approach which is suitable for analyzing datasets with fewer replicates to identify *DB* regions with high confidence. We adopt the random combination method to derive the null distributions of test statistics $T_{i}^{\left(obs\right)}$ ’s and to find the corresponding *p*-values (Figure 1C). The main idea is to re-group among the total number of *n* datasets (*n = nj + nc*) by random combination function into comparisons of two samples (*C* or *J)*, and subsequently calculate another set of r test statistics after re-grouping. The rationale of regrouping of datasets by combination to generate comparisons under the null (Figure 1C) is that the *t* statistics of *DB*s between J and C should outweigh that from the replicates of the same sample, and thus randomly switching replicates from different samples should yield the null distributions of the *t* statistics. We repeat this process *z* times, where z refers to the number of comparisons under the null hypothesis. Here we want to emphasize that we work on the unaudited column permutation, because the *t* statistics are not affected by the sequence of samples (e.g., *J* vs*. C or C* vs*. J*) or the sequences of replicates of the sample (e.g., *J1, J2* or *J2, J1* in the matrix table). Specifically, we calculate z to be half the number of different dataset combinations, which is equal to the number of all possible arrangements of datasets among *J* and *C,* keeping n_j_ and n_c_ unchanged but excluding two original configurations (*J* vs*. C* and *C* vs*. J*). Thus

$$z=\frac{C\left(n,n_{j}\right)-2}{2}=\frac{C\left(n,n_{j}\right)}{2}-1=\frac{\frac{n!}{n_{j}{!}_{*}n_{c}!}}{2}-1$$

For example, of representative datasets shown in Figure 1C, whereas *nc* = 2, *nj* = 2, *n = nc + nj* = 4, *z* will be 2 as calculated: $z=\left(C\left(4,2\right)-1\right)/2=\left(4!\right./\left(2*2!*2!\right))-1=2$ . These correspond to two comparisons under the hypothetical null (Figure 1C). For calculation of each region from a comparison under the null hypothesis, the new test statistics are denoted by $T_{l}^{\left(k\right)}$ ’s for l=1,..,q , and *k =* 1, ... , *z*. The new q∗z test statistics is used to approximate the null distribution.

The *p*-value for each row/region is subsequently defined using the following formula:

$$p_{i}=\frac{\sum_{k=1}^{z}\sum_{l=1}^{q}f\left(T_{l}^{\left(k\right)},T_{i}^{\left(obs\right)}\right)}{q*z}$$

$$f\left(T_{l}^{\left(k\right)},T_{i}^{\left(obs\right)}\right)=\left\{\begin{array}{c} 1\;if\;\left|T_{l}^{\left(k\right)}\right|>\left|T_{i}^{\left(obs\right)}\right|\\ 0\;if\;\left|T_{l}^{\left(k\right)}\right|\leq \left|T_{i}^{\left(obs\right)}\right| \end{array}\right.$$

Next, we applied the Benjamini–Hochberg correction to *p*_*i*_ (Benjamini and Hochberg, 1995) to compute adjusted *p*-values for the *i*-th row (*p*_*i-adj*_) to select statistically significant *DB* regions (Figure 1). Finally, a fold-change (FC) is calculated for *DB*s by using the average of the different groups following the equation below.

FC={IP−INc*IP−INj* if IP−INc*≥IP−INj*−1∗IP−INj*IP−INc* if IP−INc*< IP−INj*

### 3.2 CSSQ performs robust statistical analysis to identify and quantify *DB*s

#### 3.2.1 Benchmarking CSSQ performance by computational simulations

**FIGURE 3:**
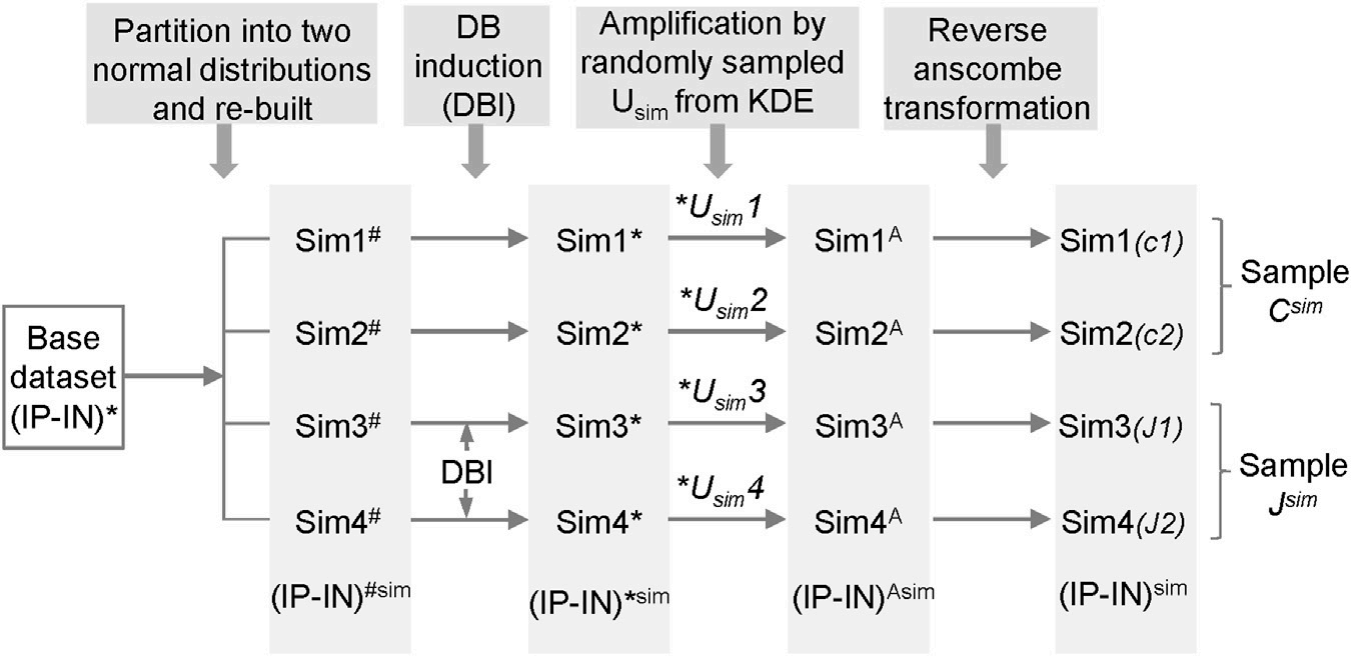
The scheme for generating simulated datasets.

Due to the absence of a gold standard for quantitative analysis of differential binding, we employed computational simulations to test CSSQ performance. Simulation studies enable induction of true positive (TP) *DB*s with varying magnitude and scope, allowing comparisons of CSSQ with parallel pipelines, CSAW and DiffBind, for benchmarking performance. We devised a scheme to create simulated datasets that resemble real datasets with true *DB* induction (*DBI*) (Figure 3, Supplementary Figure S4, Methods). For each simulated experiment, an *q ∗ n* matrix where *q* is the number of regions and *n* is the total number of datasets (*n* = 4 represented in Figure 1C) was generated, including two hypothetical replicates of the two samples (designated as *C*, *J*) for comparisons.

To generate each simulated dataset of *Sim1-4*, a base (*IP-IN*)* dataset derived from a real dataset was partitioned into two normal distributions from which parameters were extracted to re-build a simulated base dataset of *Sim1-4* ^*#*^ with randomized numbers. *DB*s were induced in *Sim3-4* ^*#*^ on randomly selected data points by changing values with varying or fixed (2–6) times the standard deviation (SD) of the corresponding cluster of the selected regions. The obtained 4 datasets, named as Sim1-4*, were then each amplified by a *U*_*sim*_ factor randomly sampled from the Kernel Density Estimate (KDE) curve fitted and based on *U* factors calculated from real ChIP-seq datasets (Supplementary Figure S2). The resulting datasets, designated as *Sim1-4* ^*A*^, were reverse Anscombe transformed to produce corresponding *Sim1-4* datasets (Figure 3). Using this approach, we produced simulated datasets with data distribution mimicking real datasets (Supplementary Figures S1, S4).

**FIGURE 4:**
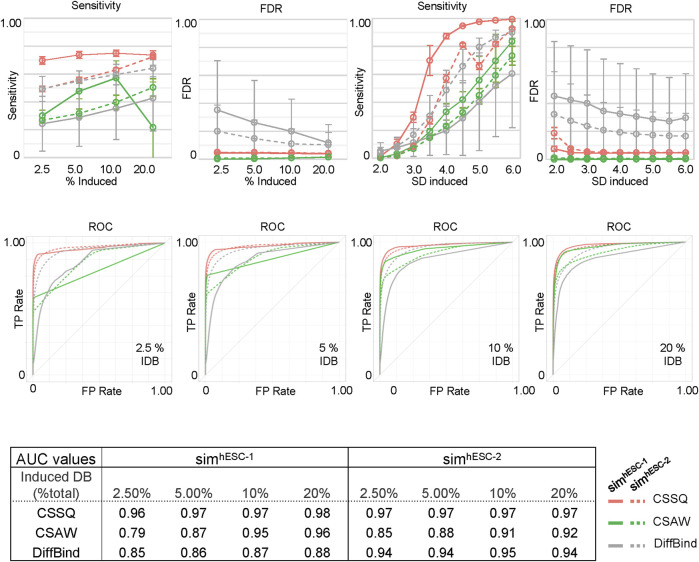
*DB* detection and quantitation on simulated datasets using CSSQ and parallel methods. Sensitivity, FDR, ROC curves and AUC values of *DB* detection are shown. Each spot averaged results from 300 simulation analyses with each simulation generated a set of four datasets based on real H3K4me3 ChIP-seq datasets of hESC-1 or hESC-2. *DB*s were induced by either alteration of variable (2–6)* SD on randomly selected data points on indicated % of data points or with fixed multiplier of SD on 2.5% data points of the data points. ROC curves and the values of Area Under the Curve (AUC) for *DB* detection of induced *DB*s by addition or reduction of values using variable SD method on randomly selected 2.5%, 5%, 10% and 20% of datapoints are shown. I*DB*: induced *DB*s. Error bars: SD.

We next scanned the performance of CSSQ, CSAW and DiffBind using *Sensitivity* (defined as true *DB*s detected/induced *DB*s), *False Discovery Rate (FDR)* (defined as false *DB*s detected/total *DB*s detected), and the receiver operating characteristic (ROC) curves as metrics. We utilized two base datasets, hESC-1 and hESC-2 H3K4me3 ChIP-seq datasets, to create two series of simulation datasets and performed simulation runs in parallel to gauge the robustness of each pipeline with datasets of different data distribution patterns (Supplementary Table S1, Supplementary Figure S1A). A total of 7,800 simulations were performed to test the effects of varying the percentages of the data points as *DBI* and of varying the magnitudes of changes of *DBI* (Figure 4, Methods). Among the three pipelines, CSSQ displayed the highest sensitivity in *DB* detection in all simulation conditions of both Sim^hESC-1^ and Sim^hESC-2^ series (Figure 4). CSSQ and CSAW had consistently low FDR, and CSSQ also exhibited superior performance in ROC curves with Area Under the Curve (AUC) higher than 0.95 in all simulations, consistently ranked the highest among three tools in all scenarios, indicating that CSSQ outperforms CSAW and DiffBind in differentiating true (induced) and false (non-induced) *DB*s (Figure 4, Supplementary Figure S5).

**FIGURE 5:**
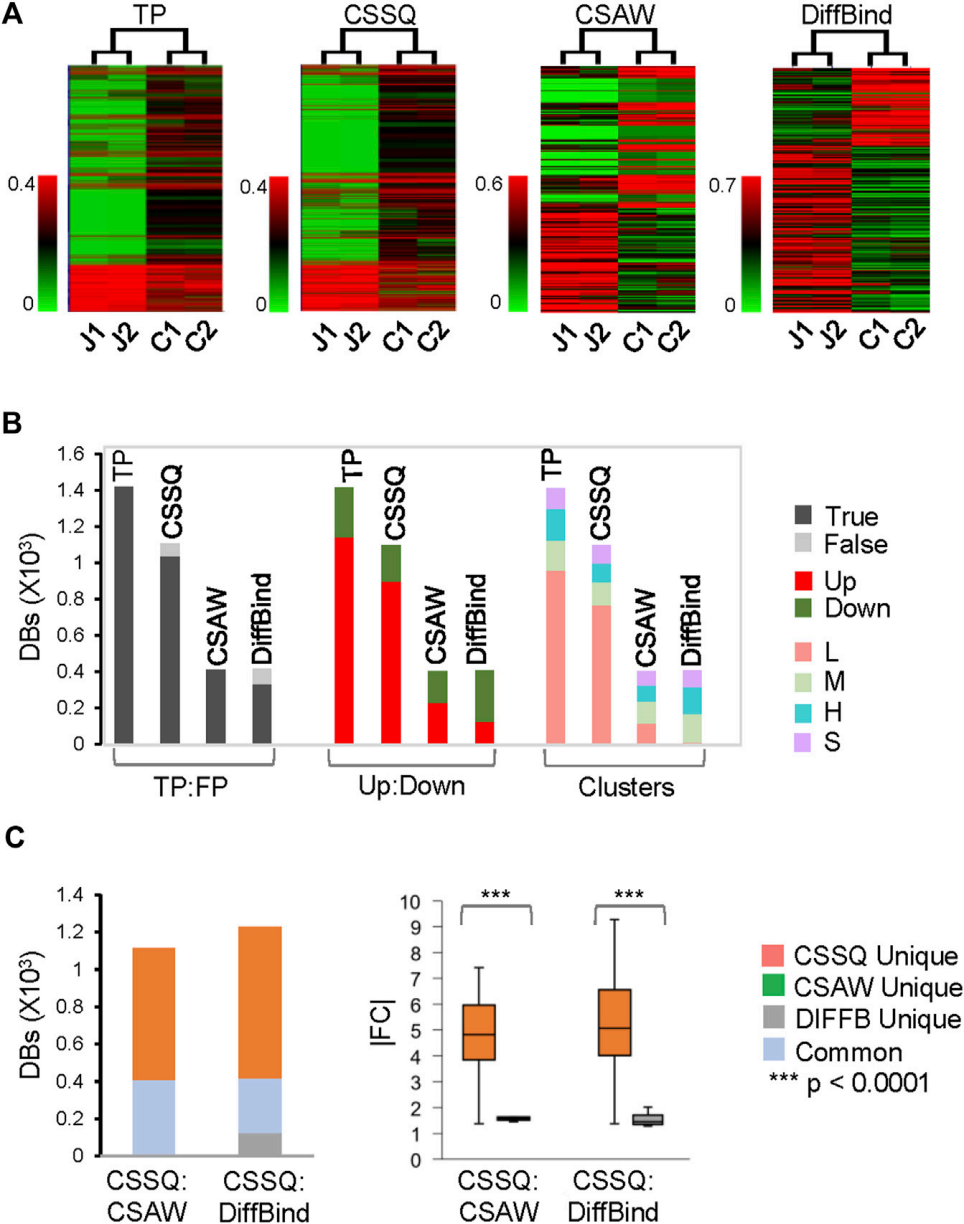
*DB* analysis on representative simulated sim^hESC-1^ datasets. 2.5% of data points were induced as *DB*s. Randomly sampled *Usim* factors from KDE were 59.2 (J1), 56.0 (J2), 53.0 (C1) and 56.0 (C2). **(A)** hierarchical clustering of *DB*s. **(B)** *DB* distributions. TP: True Positive; FP: False Positive. **(C)** *DB* comparisons by CSSQ vs. CSAW and CSSQ vs. DiffBind.

On our benchmarks, CSSQ also outperformed CSAW and DiffBind by in depth analysis of detected *DB*s against induced *DB*s (TP). We scrutinized the *DB*s detected by the three pipelines from two representative sets of 4 simulated datasets, each of sim^hESC-1^ and sim^hESC-2^ (Figure 5, Supplementary Figure S6). CSSQ *DB*s had the closest clustering pattern as that of the true *DB*s, and CSSQ consistently detected the highest number and percentage of true *DB*s with very low % of false positive calls (Figure 5A, 5B, Supplementary Figure S6). In the sim^hESC-1^ sample analysis, 1,420 *DB*s were induced with 81% being up *DB*s. CSSQ detected 1,035 of TP *DB*s, whereas CSAW and DiffBind only detected 408 and 329 TP *DB*s, supporting the superior sensitivity of CSSQ in *DB* detection (Figure 5B). Further, CSSQ detected 82% of *DB*s upregulated, closely mimicking *DBI*; whereas CSAW and DiffBind had fewer, 58% and 32%, of *DB*s as upregulated (Figure 5). *DB* partition into clusters indicated that CSSQ *DBs* had cluster distribution closely matching *DB*I while CSAW and DiffBind *DB*s deviated significantly from TP *DB*s (Figure 5)*.* Pairwise comparisons found the majority of CSAW and DiffBind *DB*s also detected by CSSQ, having 399 and 291 common *DB*s in CSSQ vs CSAW and CSSQ vs DiffBind respectively. On the other hand, a majority of CSSQ *DB*s were unique, with 706 and 894 CSSQ unique *DB*s identified from CSSQ vs CSAW and CSSQ vs*.* DiffBind, respectively. CSSQ unique *DB*s exhibited average “absolute fold changes” (|FC|) of 4.7 and 5.2, whereas unique *DB*s from CSAW (9 *DB*s) and DiffBind (123 *DB*s) only had average |FC| of 1.8 and 1.5 (Figure 5C). Similar trends were present in *DB*s detected from sim^hESC-2^ sample datasets using the three pipelines, suggesting an overall robustness of CSSQ in *DB* analysis (Supplementary Figure S6).

### 3.2.2 Analysis of real ChIP-seq datasets

We next tested CSSQ performance on real ChIP-seq datasets of H3K4me3 and H3K27me3, two characteristic histone marks with typical sharp and broad peaks, respectively (Benayoun et al., 2014; Cai et al., 2021). Toward this end, we analyzed four well-characterized cell lines, including two human cell lines of different cell types, the H1 hESC cell line and the K562 myeloid leukemia cell line, as well as two highly similar mouse ESC cell lines, the wild-type (WT) and H1c/H1d/H1e triple knockout (TKO) ESCs (Fan et al., 2005; Consortium, 2011; Geeven et al., 2015). H3K4me3 signals were analyzed for gene promoter regions flanking TSS, whereas H3K27me3 signals were compared for H3K27me3-rich regions [designated as “MRRs” or “super silencers” (Cai et al., 2021)]. The signals of H3K4me3 at gene promoters positively correlate with gene expression levels, thus transcriptome profiles of differentially expressed genes (DEGs) were included as measurement controls for *DB* detection of H3K4me3 at promoters. For H3K27me3 analysis, *DB* detection was performed for K562 MRRs, including “all K562 MRRs,” “K562 only MRRs” (K562 MRRs excluding overlapping hESC MRRs) and “K562-hESC overlapping MRRs”.

**FIGURE 6:**
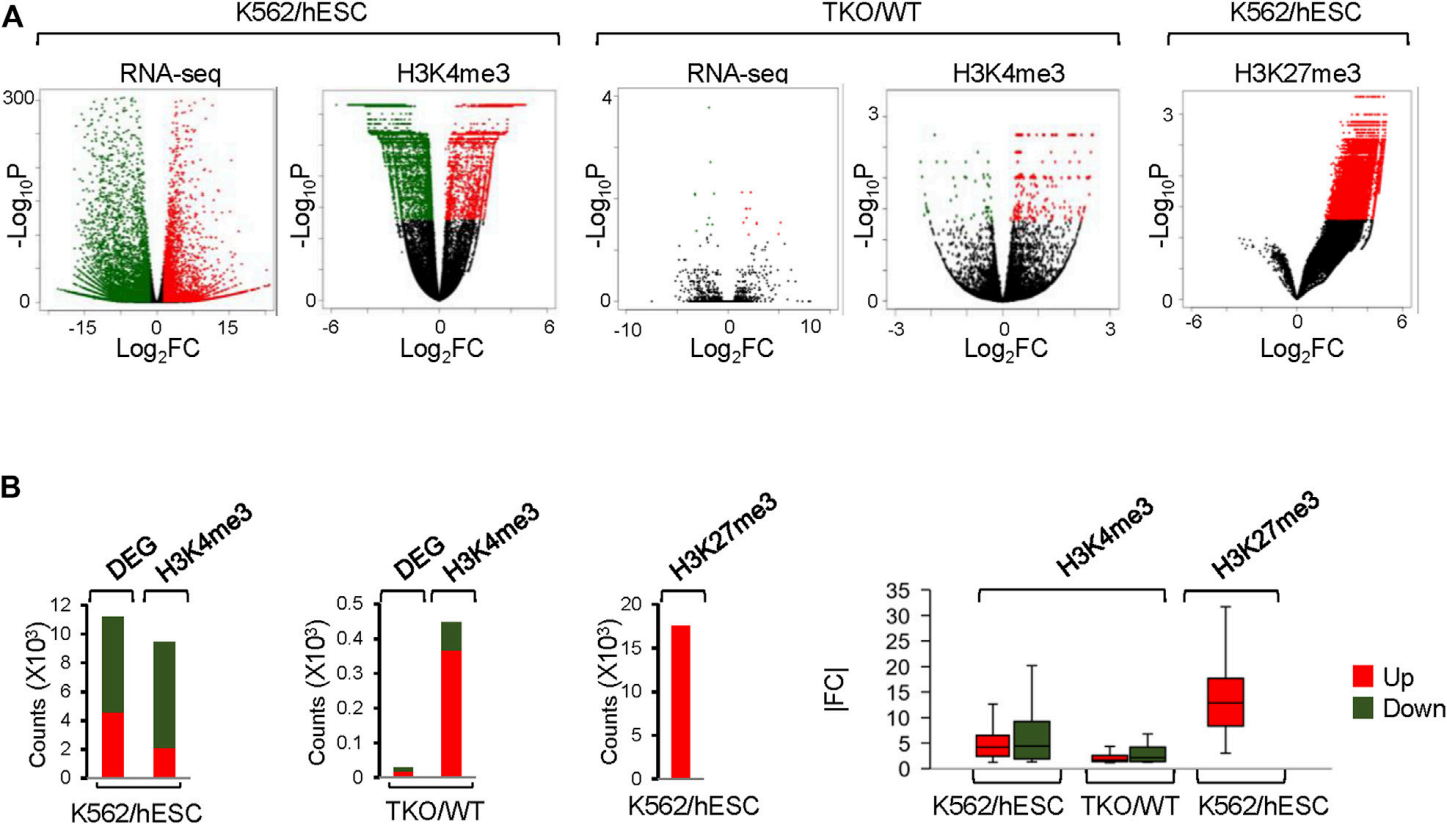
Analysis of DEGs and CSSQ *DB*s on real ChIP-seq datasets. **(A)** Volcano plots showing *DB*s and DEGs Identified in H3K4me3 and H3K27me3 datasets. Upregulated and downregulated datapoints (*p* < 0.05) are marked in red and green respectively. **(B)** bar plots of *DB* and DEG counts and |FC| of identified upregulated (Up) and downregulated (Down) *DB*s.

CSSQ identified 9,423 and 447 H3K4me3 *DB*s with in K562/hESC and TKO/WT comparisons. Such dramatic differences in the number of *DB*s detected in K562/hESC and TKO/WT comparisons mimicking the differences of DEGs in corresponding transcriptome analyses (Figure 6). hESCs and K562 cells exhibited distinctively gene expression profiles with 11,197 DEGs, whereas WT and H1 TKO ESCs had minimal gene expression changes, with only 27 DEGs of 2-fold changes from RNAseq analysis (Figure 6), consistent with previous findings (Fan et al., 2005; Geeven et al., 2015; Pan and Fan, 2016). CSSQ identified *DB*s were also in similar up/down trend to DEGs for both K562/hESC and TKO/WT comparisons, with upregulated *DB*s accounting for a minority, 22% in K562/hESC, and a majority, 82% in TKO/WT comparisons (Figure 6). CSAW detected comparable numbers of Up/Down *DB*s for K562/hESC, and DiffBind found 2,262 *DB*s for TKO/WT with only <3% being upregulated, in striking contrast to the characteristic profiles of TKO/WT DEGs of limited number and a majority (67%) as upregulated (Figure 6).

**FIGURE 7:**
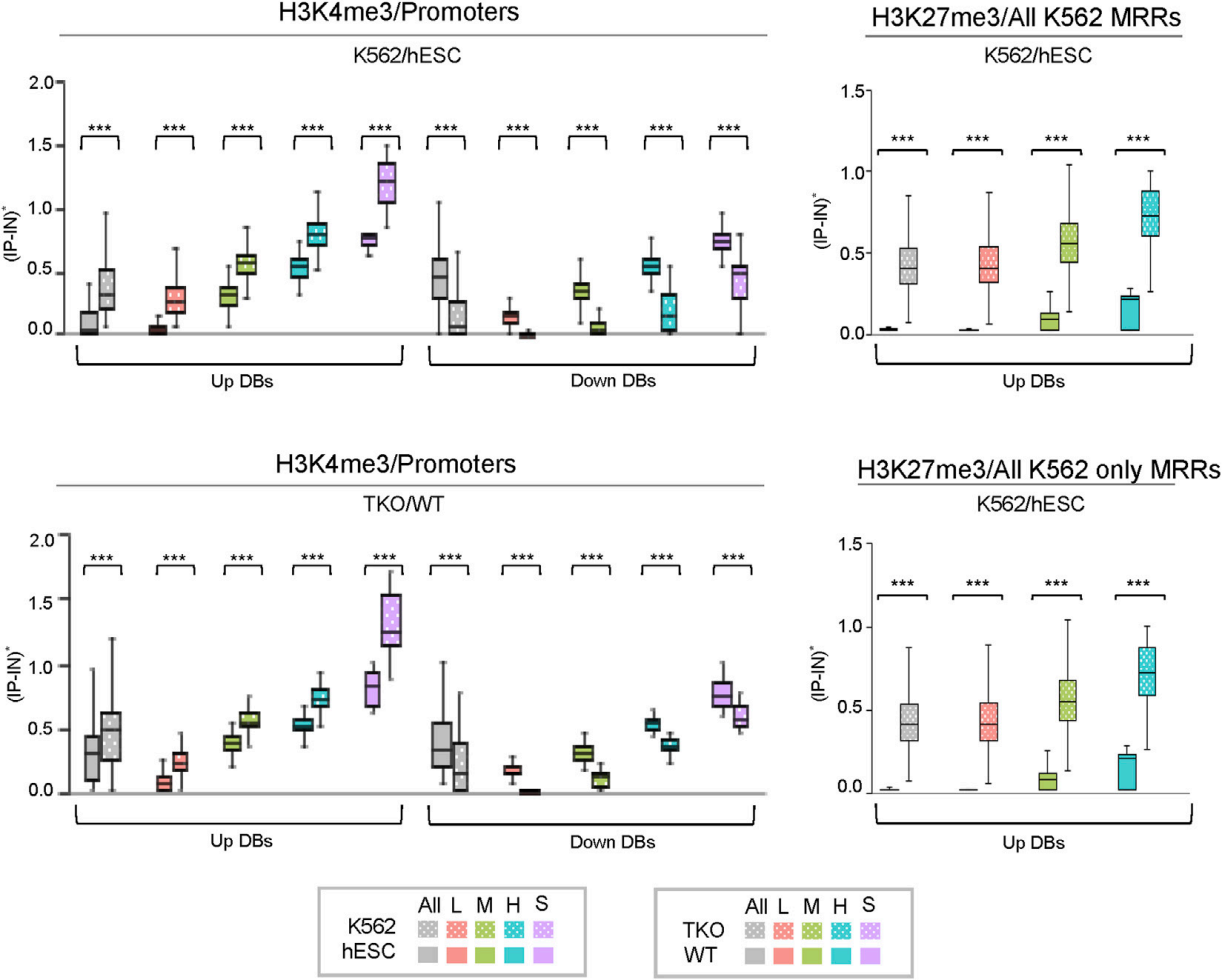
Box plots of CSSQ normalized signals for *DB*s in different clusters identified using CSSQ analysis of real ChIP-seq datasets. Left: comparisons of H3K4me3 ChIP-seq datasets from human K562 vs. hESC cells and mouse TKO vs WT cells over promoter regions (TSS ± 1 kb); Right: comparisons of H3K27me3 ChIP-seq datasets from human K562 vs. hESC cells over all K562 MRR regions and over K562 only MRR regions. ***p < 0.0001.

Metagene profiling of *IP-IN* signals of H3K4me3 CSSQ *DB*s partitioned in clusters revealed a clear difference in the signal levels of increasing signal intensity in L, M, H, and S clusters across regions flanking TSS (Supplementary Figure S7). CSSQ *DB*s in clusters also followed the trend of up/down proportion of DEGs and exhibited pronounced signal differences in normalized (*IP-IN*)* values (Figure 6, Figure 7, Supplementary Figure S8). The average |FC| for CSSQ H3K4me3 *DB*s were 6.2 in K562/hESC and 2.4 in TKO/WT (Supplementary Figure S9). The higher average |FC| in K562/hESC *DB*s than that of TKO/WT is also observed in up/downregulated *DB* groups and in each cluster, consistent with expected trend from DEG profiling (Figure 6, Supplementary Figure S9). In comparison, CSAW and DiffBind *DB*s had lower |FC| values, at 1.8 and 1.1, in TKO/WT, respectively (data not shown).

**FIGURE 8:**
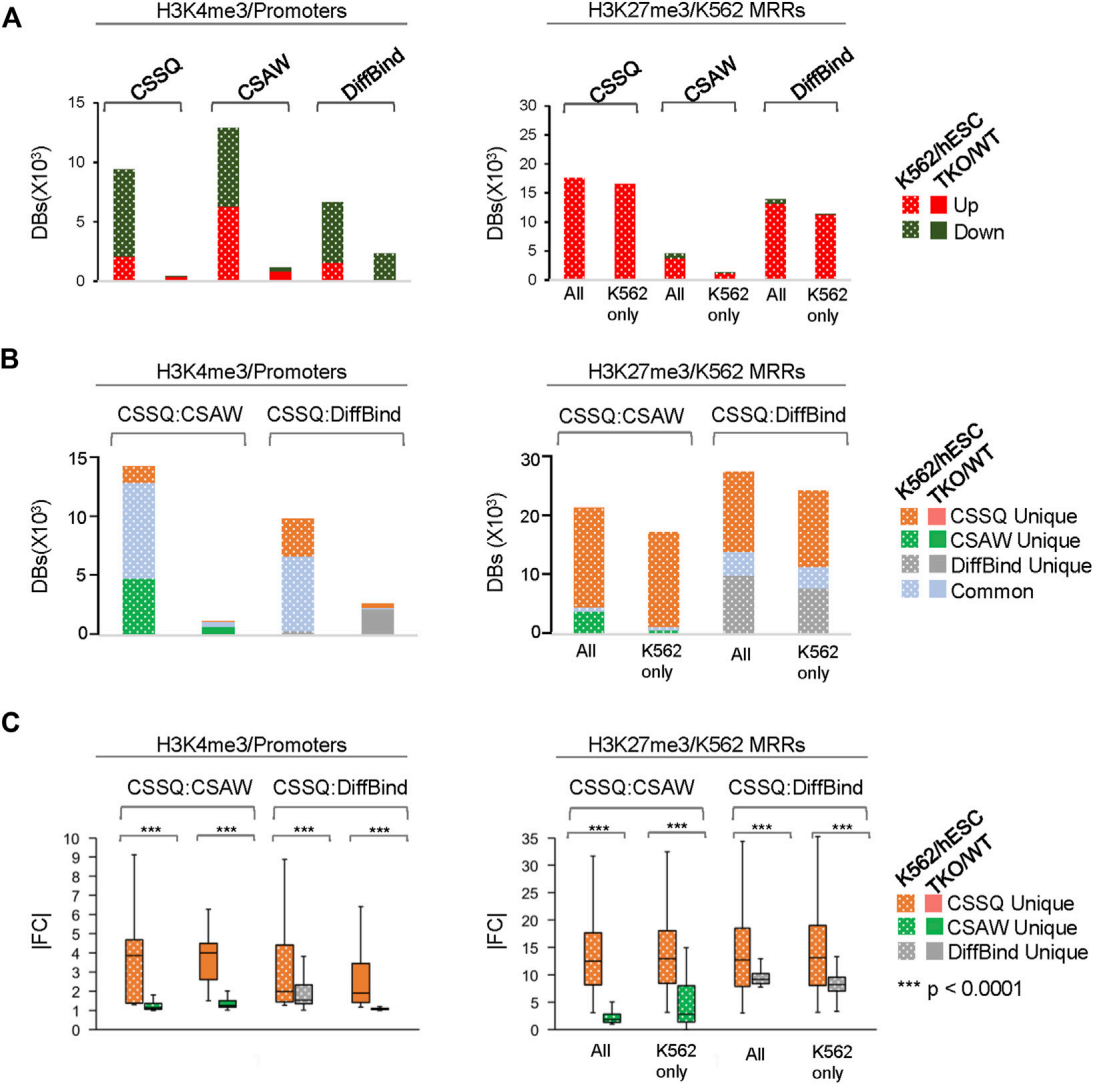
In depth analysis and comparisons of *DB*s detected by CSSQ, CSAW, and DiffBind on real ChIP-seq datasets. **(A)** Counts of Up and Downregulated *DB*s. **(B)** Counts of unique and common *DB*s identified using each tool from pair-wise comparisons. **(C)** |FC| of unique *DB*s identified using each tool from pair-wise comparisons. Labels for H3K27me3 dataset—All: all K562 MRRs; K562 only: K562 only MRRs.

CSSQ performance on *DB* detection and quantification of broad peaks such as H3K27me3 was also robust. For K562/hESC analysis, among 41,948 “all K562 MRRs” and 35,023 “K562 only MRRs”, CSSQ identified 17,552 and 16,554 *DB*s, respectively, and all (100%) of the CSSQ *DB*s were upregulated (Up *DB*s) (Figure 6B, Supplementary Figure S8), validating these regions being K562 MRRs as reported (Cai et al., 2021). The CSSQ H3K27me3 *DB*s were mostly concentrated in the “L” cluster (Supplementary Figure S8). *DB* Profiling showed a clear difference in the normalized (*IP-IN*)* signal levels of CSSQ *DB*s and the average |FC| for *DB*s identified in each cluster (Figure 7, Supplementary Figure S9). In contrast, CSAW and DiffBind found fewer *DB*s among “all K562 MRRs” and “K562 only MRRs”, and both CSAW and DiffBind had downregulated *DB*s even in “K562 only MRRs” despite these MRRs barring H3K27me3 enriched regions in hESCs (i.e., free of hESC MRRs) (Figure 8A). Hierarchical clustering for *DB*s clustered the replicates together for all three pipelines (Supplementary Figure S10).

**FIGURE 9:**
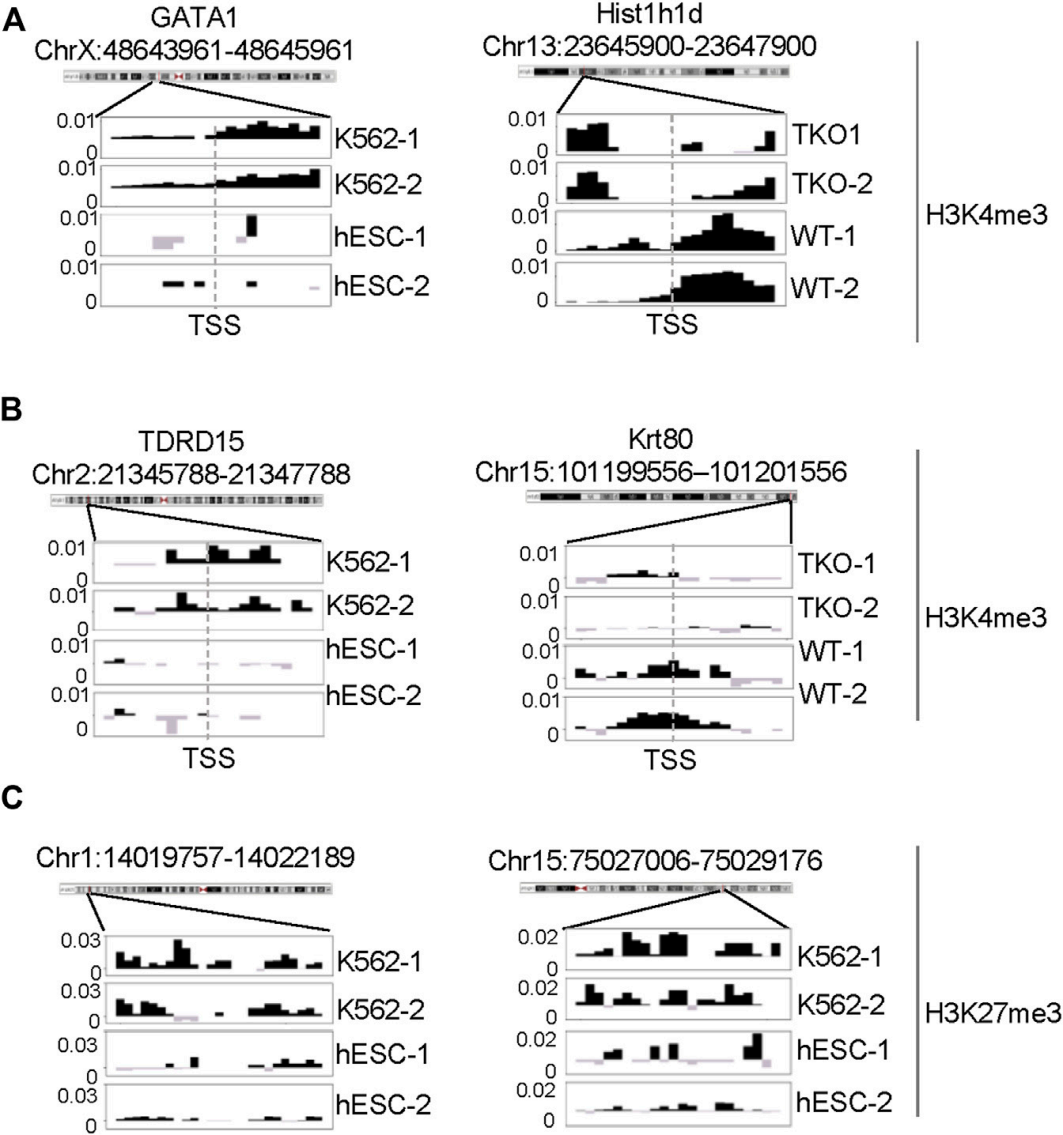
ChIP-seq signal profiles at representative loci. **(A)** H3K4me3 ChIP-seq signal profile around TSS of representative gene loci of *DB*s identified from K562 vs. hESC (left) and H1 TKO vs. WT (right) comparisons. **(B, C)** Representative H3K4me3 and H3K27me3 ChIP-seq signal profiles of unique *DB* regions identified by CSSQ in K562 vs. hESC comparisons.

To further evaluate the *DB*s detected by all three pipelines, we performed pairwise comparisons and scrutinized the common and unique *DB*s to each pipeline. For H3K4me3 K562/hESC comparison, 8,065 common *DB*s were detected in CSSQ vs CSAW and 6,243 common *DB*s were called in CSSQ vs DiffBind, whereas TKO/WT H3K4me3 analysis resulted in 400 (CSSQ vs CSAW) and 83 (CSSQ vs DiffBind) common *DB*s (Figure 8B). A representative common K562/hESC *DB* was detected at the site of GATA1, the transcription factor markedly upregulated in K562 cells, and this locus exhibited robust signal in K562 as expected (Figure 9A). H1d gene promoter region was among the 400 (CSSQ vs CSAW) common *DB*s, lacking signals in TKO cells (Figure 9A), consistent with the deletion of H1d gene and its promoter region during H1d gene targeting (Fan et al., 2001; Fan et al., 2005). DiffBind did not detect H1d as a *DB*. For H3K27me3 *DB* analysis of K562/hESC at K562 MRRs, 705 and 4,009 common *DB*s were detected in CSSQ vs. CSAW and CSSQ vs. DiffBind, respectively (Figure 8B).

The average |FC| of CSSQ unique *DB*s were statistically higher (*p* < 0.0001) than those of CSAW and DiffBind in all pair-wise comparisons (Figure 8C). Visual inspection of individual CSSQ unique *DB*s also validated *bona fide* signal enrichment of H3K4me3 and H3K27me3 peaks as the *DB* regions (Figures 9B, C). For H3K4me3 *DB*s, when compared with CSAW, unique CSSQ *DB*s had an average |FC| of 3.6 (K562/hESC) and 3.7 (TKO/WT) while that of CSAW displayed 1.4 FC (K562/hESC) and 1.5 FC (TKO/WT) (Figure 8C). Similarly, the |FC| of unique CSSQ *DB*s were significantly higher than that of DiffBind unique *DB*s (Figure 8C). The |FC| difference of unique CSSQ *DB*s was even more prominent when H3K27me3 K562/hESC *DB*s were gauged. From “all K562 MRRs” analysis, unique CSSQ *DB*s had mean |FC| at 13.6 and 13.9 as compared with that of unique *DB*s of CSAW and DiffBind at 2.6 and 7.3 from CSSQ vs. CSAW and CSSQ vs. DiffBind comparisons, respectively (Figure 8C). A similar trend was observed with *DB* analysis on “K562only MRRs” and “K562/hESC overlapping MRRs” (Supplementary Figure S11).

## 4 Discussion

ChIP-seq has revolutionized the mapping of DNA binding proteins across genome *in vivo*. The massive increase in the amount of data being generated from ChIP-seq and related methods demands robust computational tools that allow for detection and quantitation of genome-wide protein binding (Schmidl et al., 2015; Visa and Jordan-Pla, 2018). However, powerful pipelines capable of direct quantitation of ChIP-seq signals across different datasets are in short supply (Steinhauser et al., 2016; Nakato and Sakata, 2021). By employing sophisticated statistical approaches, we developed CSSQ, an innovative ChIP-seq signal quantifier that is capable of detection and quantitation of interesting regions with high confidence and sensitivity.

CSSQ fits a Gaussian mixture model to the transformed data instead of fitting a single distribution such as the negative binomial distribution to the raw count data. It utilizes a combination of Anscombe transformation, *k*-means clustering, and estimated maximum normalization to minimize noise and bias from experimental variations. Anscombe transformation effectively converts ChIP-seq raw data from an approximate mixture of Poisson distributions (Supplementary Figure S12) into Gaussian mixture models. Our choice of k clustering into 4 groups here is based on extensive tests and analyses. We find that k = 4 in general balances the goodness-of-fit and model complexity of datasets tested. As illustrated in the representative scree plots generated on “Variance Explained” (within-in cluster variance) and “Adjusted R-squared” (between-cluster variance) with respect to different number of clusters (Supplementary Figure S13), based on the elbow method, k = 4 represents a bend position and a robust number of clusters, e.g., the improvement from k-3 to k = 4 is still relatively large (4%), while the improvement from k = 4 to k = 5 (2%) turns to relatively small. In addition, this clustering partitions the data points into 4 well-defined, biologically meaningful data groups representing low (L), medium (M), high (H), super high (S) signals, respectively.

To statistically test significance and *DB* calling, CSSQ utilizes a non-parametric approach and incorporates comparisons under the null hypothesis by re-shuffling datasets among different groups to perform robust statistical tests to account for fewer replicates of ChIP-seq datasets of the same biological samples. It reports an adjusted *p*-value and a fold-change (FC) for all pre-defined regions. The normalization method adopted in CSSQ and the generation of comparisons under the null hypothesis by re-shuffling of datasets for *DB* calling are novel applications of mathematical treatments to ChIP-seq datasets. These approaches employed by CSSQ play a critical role in reducing false *DB* calls that may arise due to experimental noises and biases.

Using simulation studies and quantitative metrics, we benchmarked CSSQ with the CSAW and DiffBind pipelines. CSSQ identified induced *DB*s with a low FDR and high sensitivity under all simulation scenarios. While CSAW had a slightly lower FDR than CSSQ, its sensitivity was not consistent and much lower than CSSQ in nearly all simulations (Figure 4). Even though DiffBind occasionally had similar sensitivity as CSSQ in *DB* calls, such as in sim^hESC-2^ simulations, it consistently had a higher FDR (Figure 4). CSSQ exhibited the highest power and robustness in distinguishing between true and false *DB*s, as evidenced from its ROC curves and the highest AUCs in all simulations. Further, in our in-depth analysis of a simulated dataset, unique *DB*s identified by CSSQ had a higher |FC| with statistical significance (*p* < 0.0001) when compared to those of CSAW and DiffBind, suggesting that unique *DB*s of CSSQ are more likely to be true *DB*s than those of CSAW and DiffBind (Figure 5C). The results were consistent in both simulation studies, one analyzing the performance on different percentages of true *DB*s and the other on different magnitudes of signal differences in *DBI*. The results from these simulations demonstrate the robustness of the CSSQ pipeline.

CSSQ also outperforms parallel pipelines in benchmarking exercises using real ChIP-seq datasets. K562 human multipotent leukemia cell line and H1 human pluripotent embryonic stem cell line are two distinct cell types with 11,197 DEGs that accounts for nearly 20% of all genes in the human genome. While ChIP-seq analysis of promoter H3K4me3 reflected this well with all three tools identifying thousands of potential *DB*s. including various cell specific marker genes, unique *DB*s from CSSQ had a higher |FC| when compared with those from CSAW and DiffBind. In addition to comparison test using ChIP-seq datasets from entirely different cell types, we also scrutinized *DB*s detected by the three pipelines on datasets from two highly similar cell lines, mouse H1 TKO and WT ESCs that had very limited gene expression changes at undifferentiated states (Fan et al., 2005). Transcriptome analysis of RNAseq datasets from TKO/WT comparisons only identified 27 statistically significant DEGs (Geeven et al., 2015), and CSSQ had the least number of *DB*s from analysis of the TKO/WT H3K4me3 ChIP-seq datasets among the three pipelines (Figures 6, 8).

To robustly compare the performance of the pipelines on different signal profiles, we further benchmarked using H3K27me3 ChIP-seq data which exhibit a broad signal profile. Utilizing H3K27me3-rich regions (MRR) in K562 and hESC (Cai et al., 2021), again we found that CSSQ has a higher sensitivity in identifying *DB*s than CSAW and DiffBind (Figures 6, 8).

CSSQ and DiffBind were designed to analyze predefined regions of interests. DiffBind uses statistical routines developed for RNAseq (DESeq2 (Love et al., 2014) and EdgeR (Robinson et al., 2010)) to identify significant *DB* regions (Ross-Innes et al., 2012). CSAW was primarily designed for genome-wide *de novo* detection of *DB* regions between samples using statistical tests implemented in edgeR. CSAW, like DiffBind, utilizes statistical testing methods that were developed for differential gene expression analysis in EdgeR package (Robinson et al., 2010; Lun and Smyth, 2016). The inherent difference between the data distribution of ChIP-seq and RNAseq datasets and the use of control libraries for background correction in ChIP-seq make it less ideal to apply the same statistical approach for RNAseq on ChIP-seq datasets (Figure 1B, Supplementary Figures S1, S14).

In summary, the CSSQ pipeline is a statistically robust pipeline to perform differential binding analysis on pre-defined regions of interest across ChIP-seq samples. It enables quantitative analysis of ChIP-seq data by utilizing statistically sound methods to normalize for experimental biases, control false discovery rate and provide high confidence *DB* calls and quantification.

## Data Availability

CSSQ is implemented as an R package available in open-source form at http://bioconductor.org/packages/release/bioc/html/CSSQ.html and https://github.com/fan-lab-gatech. The datasets used in this study can be found in online repositories. The names of the repository/repositories and accession number(s) can be found in the article/Supplementary Material.
