# Supplementary material for: CSSQ: a ChIP-seq signal quantifier pipeline
Source: Front Cell Dev Biol. 2023 May 25;11:1167111. doi: 10.3389/fcell.2023.1167111 (PMC10248417; doi:10.3389/fcell.2023.1167111)
Supplement: Supplementary file 1 [file DataSheet1.pdf]

Supplementary Material

CSSQ: A ChIP-Seq Signal Quantifier Pipeline

Ashwath Kumar[1], Michael Y. Hu[2,±], Yajun Mei[3,4], Yuhong Fan[1,4,*]

[1] School of Biological Sciences, Georgia Institute of Technology, Atlanta, Georgia 30332, USA;
[2] Department of Computer Science, Princeton University, Princeton, NJ 08540, USA; ±Present Address: Center for Data Science, New York University, New York, NY 10011, USA
[3] H. Milton Stewart School of Industrial and Systems Engineering, Georgia Institute of Technology, Atlanta, Georgia 30332, USA;
[4] Parker H. Petit Institute for Bioengineering and Bioscience, Georgia Institute of Technology, Atlanta, Georgia 30332, USA

*** Correspondence:** Corresponding author: yuhong.fan@biology.gatech.edu

Contents

Supplementary page list…………………………………………………………………………1

Supplementary Table S1……………………..………………………………………….......2

Supplementary Figures…………………………………………………………………….3-16
Figures S1-S14

Supplementary References…………...………………………………………………………17

SUPPLEMENTARY TABLE

Supplementary Table S1. Summary of datasets

Dataset		Cell type	Species	Replicates	GEOID	GEO series (Downloaded files/genomes aligned)	Reference
H3K4me3 ChIPseq	IP	hESC	Human	1, 2	GSM733657	GSE29611 (BAM/hg19)	Consortium, 2011
	Input	hESC	Human	1, 2	GSM733770		
	IP	K562	Human	1, 2	GSM733680		
	Input	K562	Human	1, 2	GSM733780		
	IP	HSMM	Human	1, 2	GSM733637		
	Input	HSMM	Human	1, 2	GSM733663		
	IP	HUVEC	Human	1, 2	GSM733673		
	Input	HUVEC	Human	1, 2	GSM733715		
	IP	HeLa-S3	Human	1, 2	GSM733682		
	Input	HeLa-S3	Human	1, 2	GSM733659		
	IP	WT	Mouse	1	GSM1954949	GSE75426 (FASTQ/mm9)	Geeven,et al., 2015
				2	GSM1954950		
	Input	WT	Mouse	1	GSM1954943		
				2	GSM1954944		
	IP	H1 TKO	Mouse	1	GSM1954951		
				2	GSM1954952		
	Input	H1 TKO	Mouse	1	GSM1954947		
				2	GSM1954948		
	IP	Heart	Mouse	1, 2	GSM722694	GSE29184 (BAM/mm9)	Shen, et al., 2012
	Input	Heart	Mouse	1, 2	GSM722697		
	IP	Bone Marrow	Mouse	1, 2	GSM722638		
	Input	Bone Marrow	Mouse	1, 2	GSM722640		
	IP	Cerebellum	Mouse	1, 2	GSM722665		
	Input	Cerebellum	Mouse	1, 2	GSM722667		
H3K27me3 ChIPseq	IP	hESC	Human	1, 2	GSM733748	GSE29611 (BAM/hg19)	Consortium, 2011
	Input	hESC	Human	1, 2	GSM733770		
	IP	K562	Human	1, 2	GSM733658		
	Input	K562	Human	1, 2	GSM733780		
RNAseq		hESC	Human	1	GSM5331257	GSE175297 (FASTQ/hg19)	Consortium, 2011
				2	GSM5331258		
		K562	Human	1	GSM5330997	GSE175163 (FASTQ/hg19)	
				2	GSM5330998		
		WT ESC	Mouse	1	GSM1954926	GSE75426 (FASTQ/mm9)	Geeven,et al., 2015
				2	GSM1954927		
		H1 TKO ESC	Mouse	1	GSM1954928		
				2	GSM1954929		

SUPPLEMENTARY FIGURES

Supplementary Figure S1

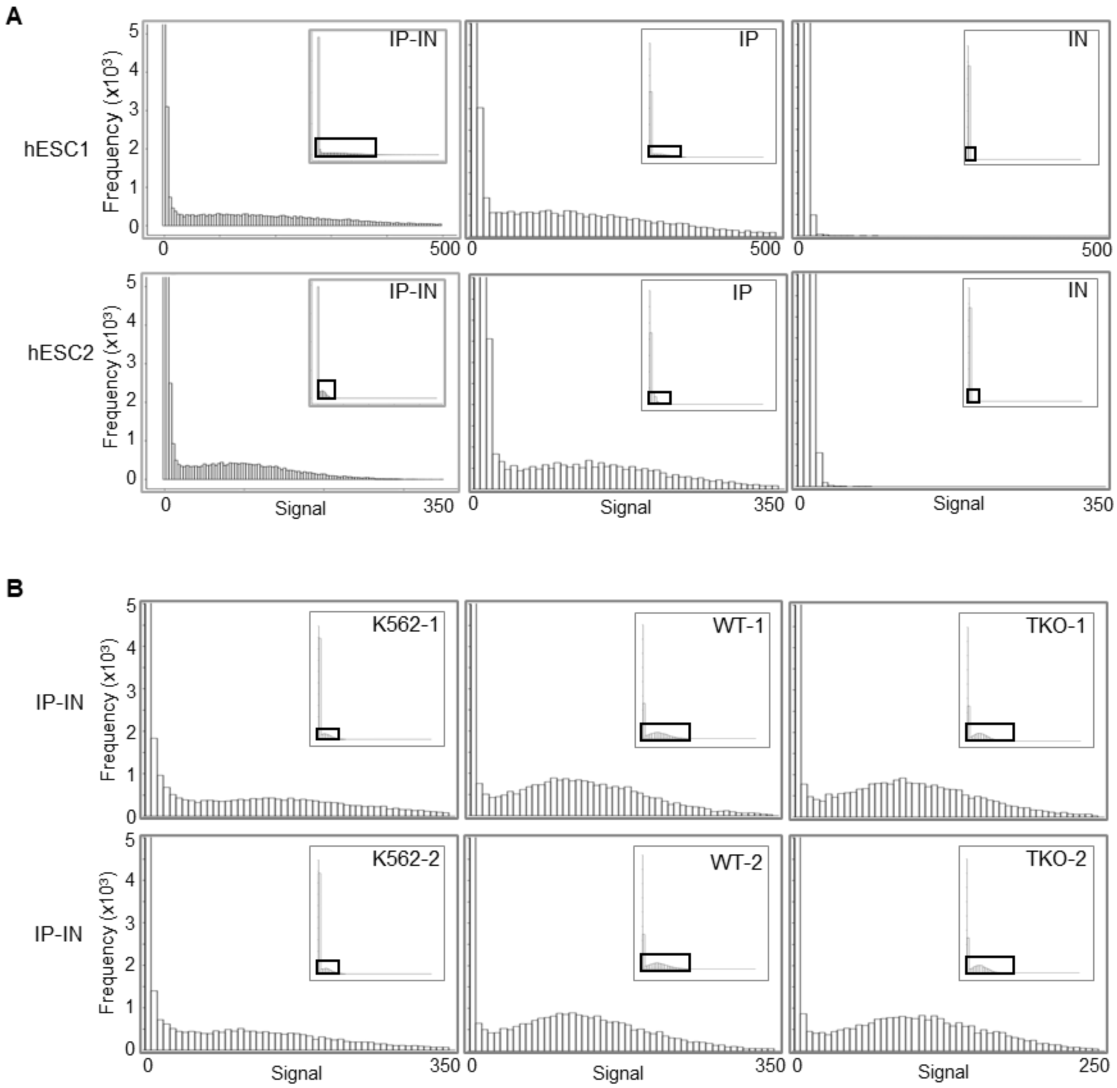

Supplementary Figure S1. Data distribution of representative H3K4me3 ChIP-seq datasets. (A) Zoomed in histograms of IP-IN, IP, IN signals from datasets of hESC-1 and hESC-2 with zoomed out histograms as insets are shown. (B) Zoomed in histograms of IP-IN signals from replicate datasets of K562, WT and H1 TKO mESCs with zoomed out histograms as insets are shown.

Supplementary Figure S2

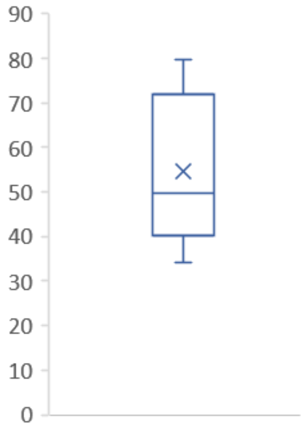

Supplementary Figure S2. Data distribution of U factors of real ChIP-seq datasets. Box plots of calculated U values of 20 H3K4me3 ChIP-seq datasets are shown.

Supplementary Figure S3

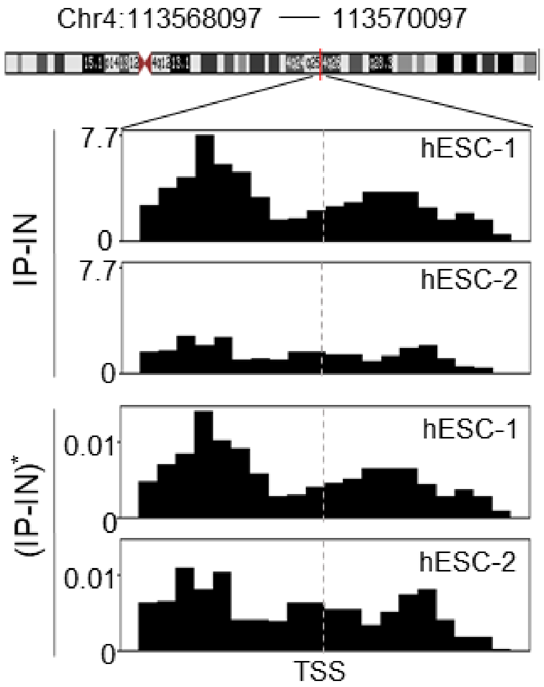

Supplementary Figure S3. Representative H3K4me3 ChIP-seq signal profiles of replicate datasets of hESCs before and after CSSQ normalization. The profiles of (IP-IN) and (IP-IN)* signals of hESC replicates at indicated genomic region are shown.

Supplementary Figure S4

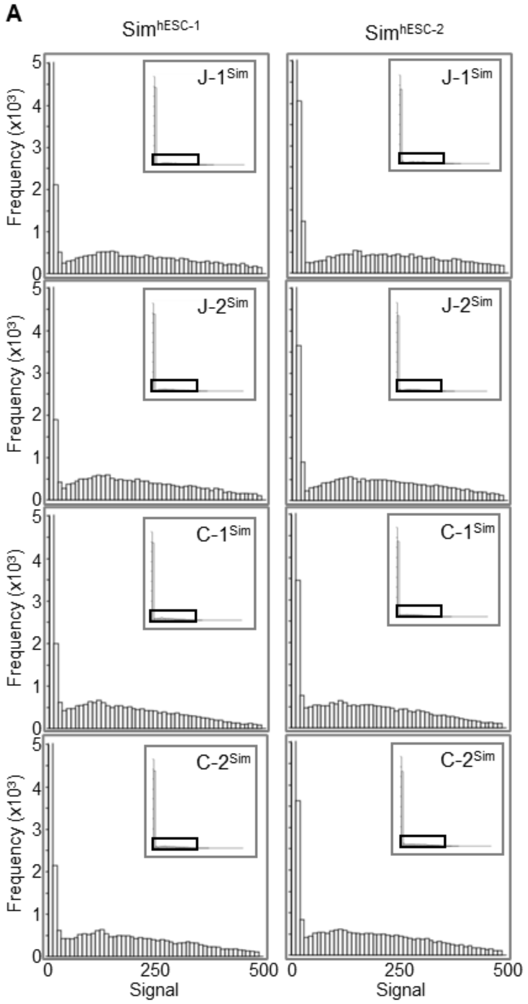

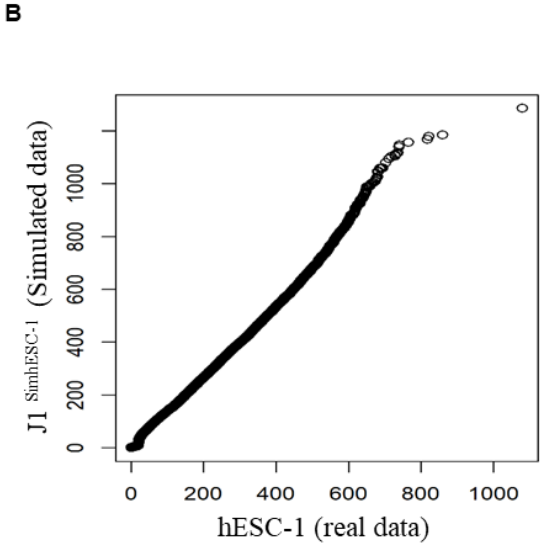

C

Data scale	Comparisons with hESC-1 real Dataset	Pearson's correlation coefficients			
		J1	J2	C1	C2
Raw data	Sim-1^{hESC-1}	0.999	0.988	0.989	0.987
	Sim-2^{hESC-1}	0.998	0.990	0.983	0.979
Transformed data	Sim-1^{hESC-1}	0.999	0.994	0.990	0.985
	Sim-2^{hESC-1}	0.997	0.998	0.983	0.976

Supplementary Figure S4. Data distribution of representative simulated datasets mimics that of the real ChIP-seq datasets. (A) Zoomed in histograms of (IP-IN) signals from representative simulated datasets based on hESC-1 and hESC-2 H3K4me3 ChIP-seq datasets with zoomed out histograms as insets are shown. (B) Representative scatter plot of hESC-1 real dataset and J1 data of a representative simulated dataset SimhESC-1. (C) Summary of Pearson's correlation coefficients of comparisons between hESC-1 real ChIP-seq dataset with corresponding representative simulated datasets.

Supplementary Figure S5

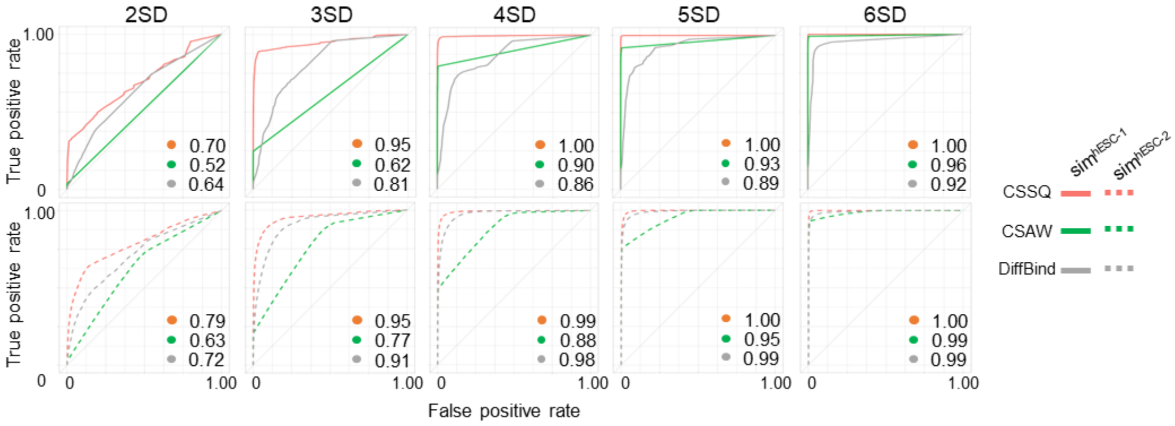

Supplementary Figure S5. ROC curves for DB detection using CSSQ and parallel methods on simulated datasets. DBs were induced on 2.5% of the data points over increasing magnitudes of differences using fixed multiplier (2-6) times of SD of the corresponding cluster as indicated. The values of area under the curve (AUC) from each pipeline are listed in the plots.

Supplementary Figure S6

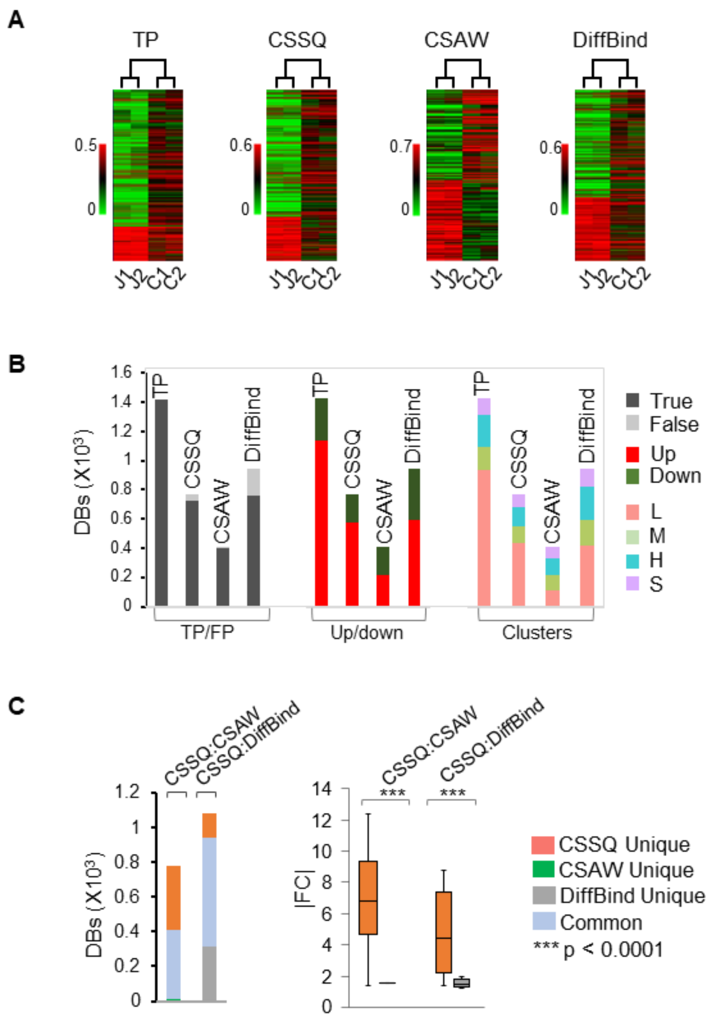

Supplementary Figure S6. DB analysis on representative simulated sim^{hESC-2} datasets. 2.5% of data points were induced as DBs. *Usim* factors applied were 59.2 (J1), 56.0 (J2), 53.0 (C1) and 56.0 (C2). (A) hierarchical clustering of DBs. (B) DB distributions. (C) DB comparisons by CSSQ *vs.* CSAW and CSSQ *vs.* DiffBind. TP: True Positive; FP: False Positive.

Supplementary Figure S7

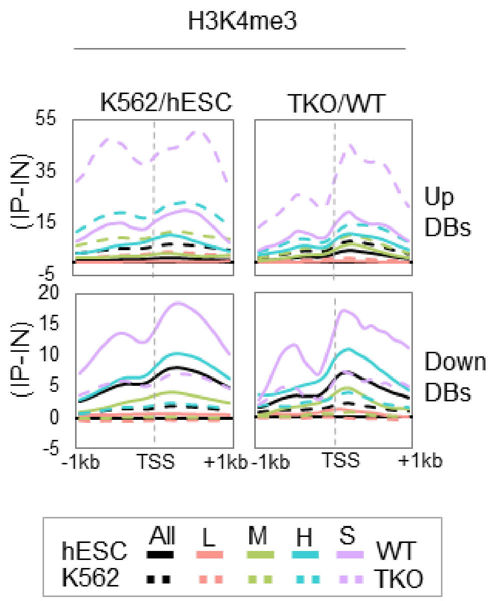

Supplementary Figure S7. Metagene plots profiling average H3K4me3 signals across promoter regions (TSS +/- 1kb) of DBs in different clusters identified using CSSQ from comparisons of H3K4me3 ChIP-seq datasets from human K562 *vs* hESC cells and mouse TKO *vs* WT cells.

Supplementary Figure S8

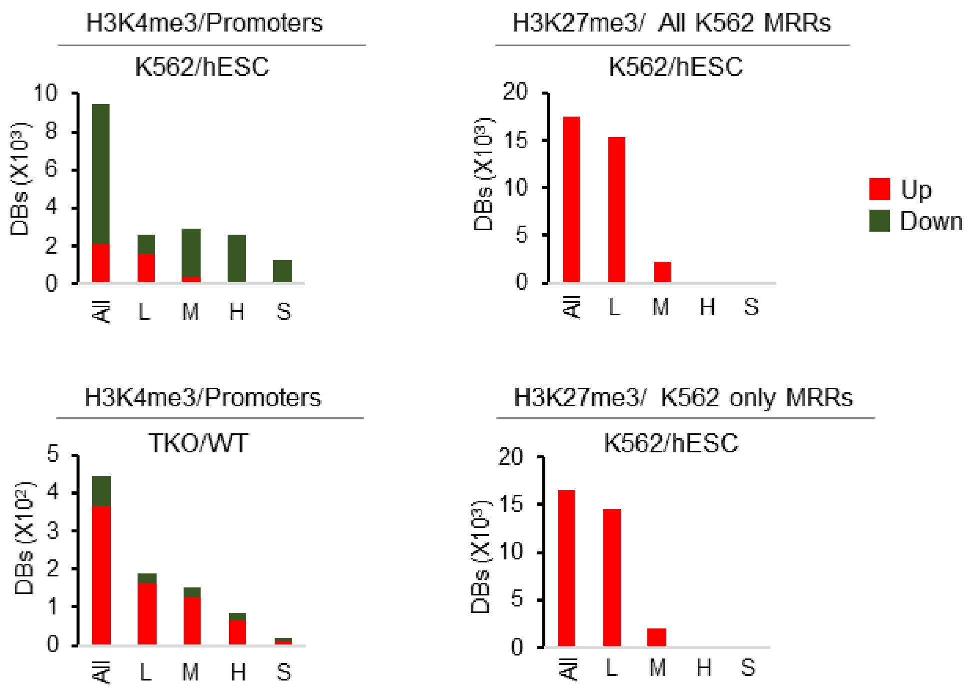

Supplementary Figure S8. Bar plots of Up/down DBs identified by CSSQ on real ChIP-seq datasets in each cluster. DBs were identified using CSSQ from comparisons of H3K4me3 ChIP-seq datasets from human K562 *vs* hESC cells (top left) and mouse TKO *vs* WT cells (bottom left) at promoter regions and H3K27me3 ChIP-seq datasets from human K562 *vs* hESC cells at "All K562 MRRs" (top right) and "K562 only MRRs" (bottom right) regions.

Supplementary Figure S9

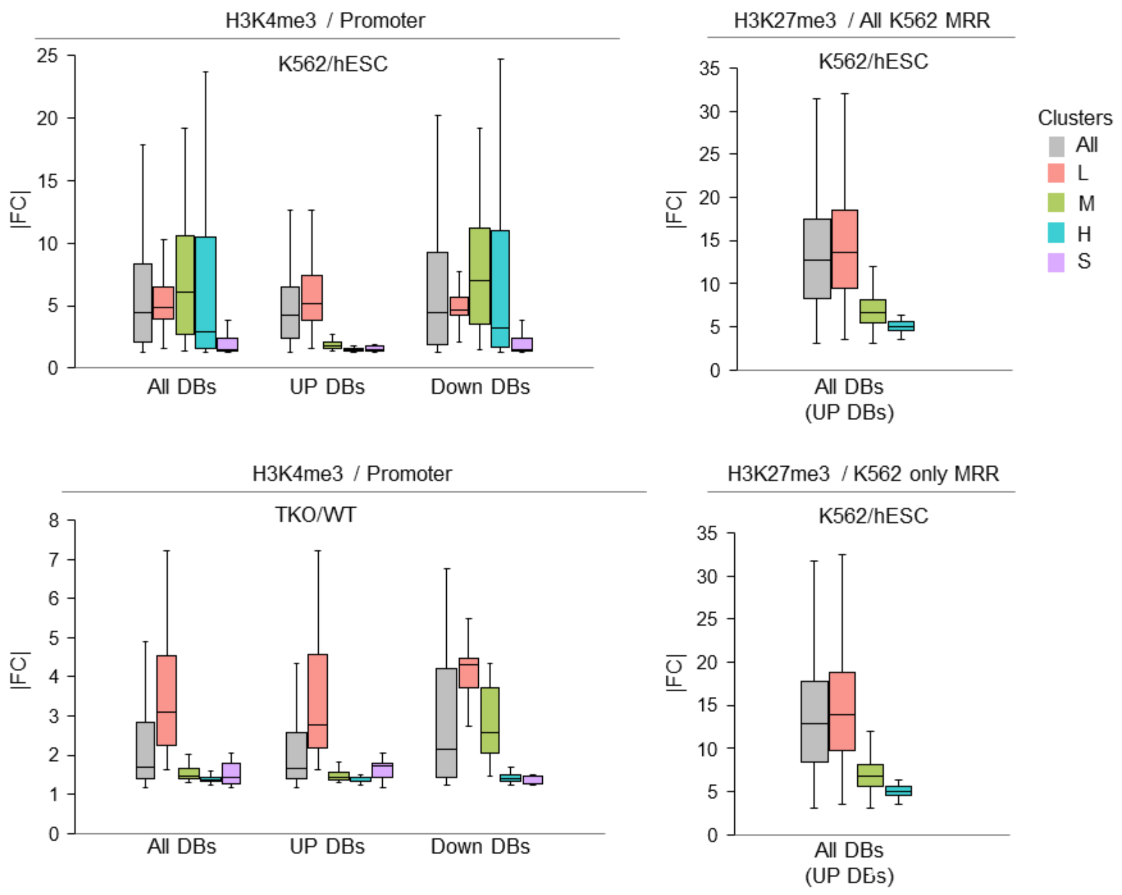

Supplementary Figure S9. Boxplots of absolute fold changes of DBs identified by CSSQ in different clusters on real ChIP-seq datasets. Left: DBs detected on H3K4me3 ChIP-seq datasets from human K562 vs hESC cells and mouse TKO vs WT cells over promoter regions (TSS +/- 1kb). Right: DBs detected from H3K27me3 ChIP-seq dataset (K562/hESC) over “all K562 MRRs” regions and “K562 only MRRs” regions.

Supplementary Figure S10

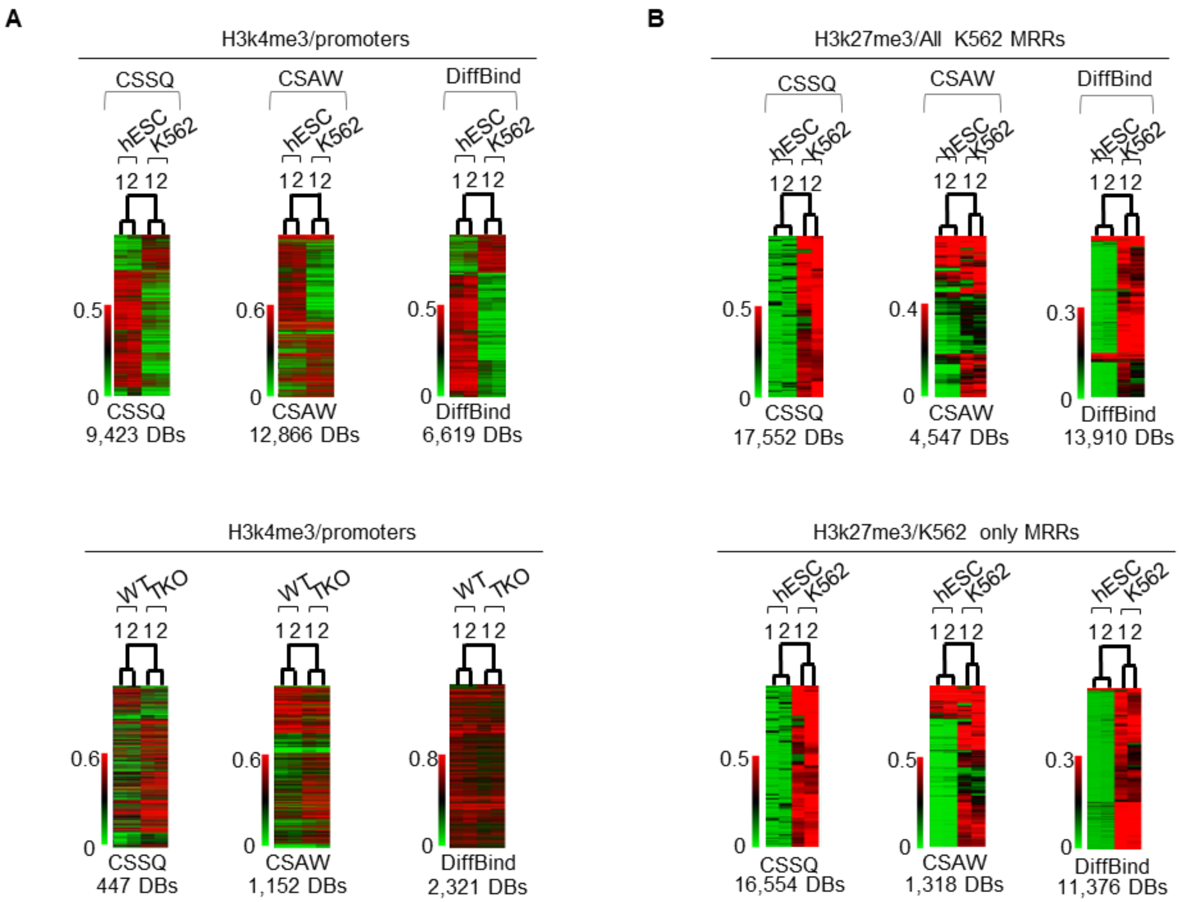

Supplementary Figure S10. Hierarchical clustering of DBs identified by CSSQ, CSAW and DiffBind on real ChIP-seq datasets. (A) Heatmaps of DBs identified from H3K4me3 ChIP-seq datasets comparisons of K562 vs. hESC (top) and TKO vs. WT (bottom) over promoter regions. (B) Heatmaps of DBs identified from H3K27me3 ChIP-seq datasets comparisons of K562 vs. hESC over “all K562 MRRs” (top) and “K562 only MRRs” regions (bottom).

Supplementary Figure S11

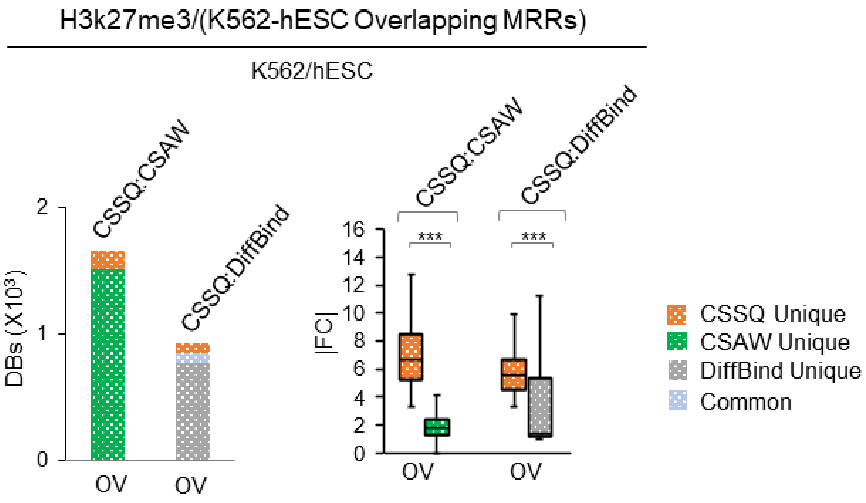

Supplementary Figure S11. CSSQ *vs.* CSAW and CSSQ *vs.* DiffBind pair-wise comparisons of H3K27me3 K562/hESC DBs from K562-hESC Overlapping MRRs. OV: overlapping regions of "All K562 MRRs" and "All hESC MRRs".

Supplementary Figure S12

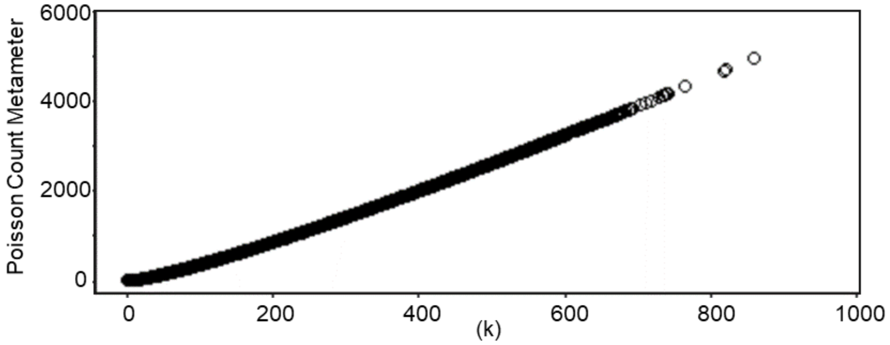

Supplementary Figure S12. Representative Poissonness plot of the raw real ChIP-seq dataset. Poissonness plot (Hoaglin, 1980;Hoaglin and Tukey, 1985) of the real hESC-1 H3K4me3 ChIP-seq dataset was generated by plotting "Poisson Count Metameter" (defined as "$\log(N_k/N)+\log(\text{factorial}(k))$") against "k", where ($N_k$ /N) denotes the fraction of observed data which take value of k after rounding for k=0,1,2,…. At larger k values, the line shows a linear trend, while at smaller k values, there is a noticeable bend or flatness in the initial segment of the line, indicating a potential mixture of Poisson distributions.

Supplementary Figure S13

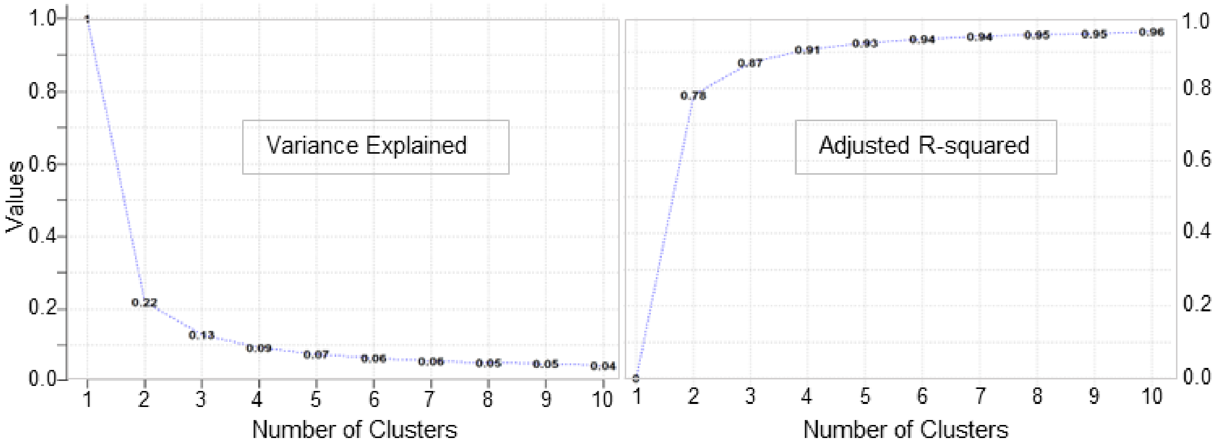

Supplementary Figure S13. "Variance Explained" (within-in cluster variance) and "Adjusted R-squared" (between-cluster variance) with respect to different number of clusters. Representative scree plots of "Variance Explained" (defined as the ratio between the sum of the within-cluster-sum-of-squares and the total sum of squares) and "Adjusted R-squared" (the adjusted R^2 statistic, defined as the between-cluster sum of squares divided by the total sum of squares) were generated using the real hESC1 H3K4me3 ChIP-seq dataset and R package "ClusterR".

Supplementary Figure S14

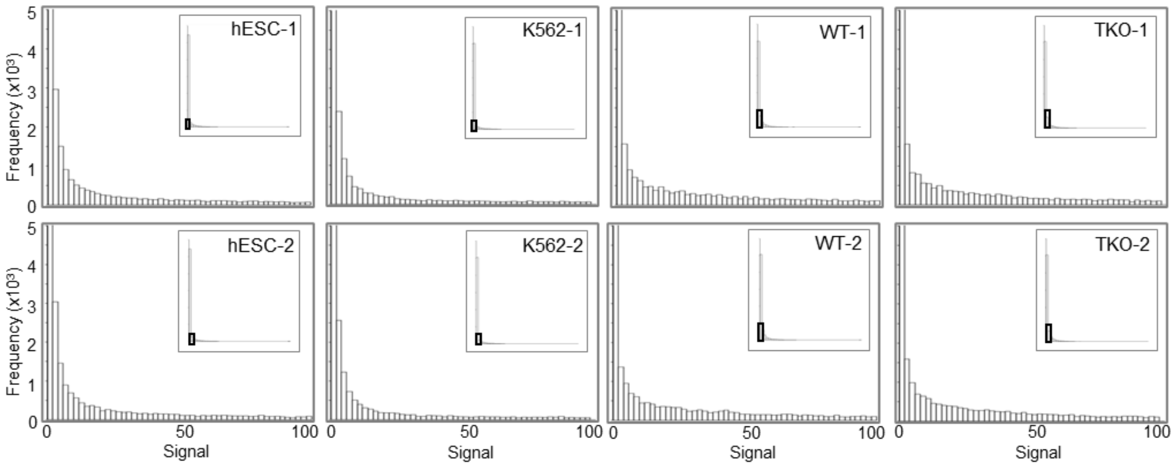

Supplementary Figure S14. Data distribution of representative replicate RNA-seq datasets of hESC, K562, and mESC (WT and H1 TKO) cells. Zoomed in histograms of RNA-seq signals with zoomed out histograms as insets are shown.

SUPPLEMENTARY REFERENCES

Consortium, E.P. (2011). A user's guide to the encyclopedia of DNA elements (ENCODE). *PLoS Biol* 9, e1001046.

Geeven, G., Zhu, Y., Kim, B.J., Bartholdy, B.A., Yang, S.M., Macfarlan, T.S., Gifford, W.D., Pfaff, S.L., Verstegen, M.J., Pinto, H., Vermunt, M.W., Creyghton, M.P., Wijchers, P.J., Stamatoyannopoulos, J.A., Skoultchi, A.I., and De Laat, W. (2015). Local compartment changes and regulatory landscape alterations in histone H1-depleted cells. *Genome Biol* 16, 289.

Hoaglin, D.C. (1980). A Poissonless Plot. *American Statistician* 34, 146-149.

Hoaglin, D.C., and Tukey, J.W. (1985). "Checking the Shape of Discrete Distributions", in *Exploring Data Tables, Trends and Shapes*, ed. F.M. D. C. Hoaglin, And J. W. Tukey. (New York: John Wiley & Sons), pp. 345-416.

Shen, Y., Yue, F., Mccleary, D.F., Ye, Z., Edsall, L., Kuan, S., Wagner, U., Dixon, J., Lee, L., Lobanenkov, V.V., and Ren, B. (2012). A map of the cis-regulatory sequences in the mouse genome. *Nature* 488, 116-120.
